# Microbial community composition and diversity in nodules and rhizosphere soil of bitter white lupine (*Lupinus albus* L.) and rhizosphere soil of triticale (*×Triticosecale Wittmack*)

**DOI:** 10.3389/fmicb.2026.1810398

**Published:** 2026-06-24

**Authors:** Mulugeta Aytenew, Aregu Amsalu Aserse, Petri Penttinen, Seyed Abdollah Mousavi, Melkamu Alemayehu, Kristina Lindström, Enyew Adgo

**Affiliations:** 1 Department of Natural Resource Management, College of Agriculture and Environmental Sciences, Bahir Dar University, Bahir Dar, Ethiopia; 2 Department of Plant Science, College of Agriculture and Natural Resources, Debre Markos University, Debre Markos, Ethiopia; 3 Ecosystems and Environment Research Programme, Faculty of Biological and Environmental Sciences, University of Helsinki, Helsinki, Finland; 4 Sichuan Agricultural University, Ya'an, China; 5 Department of Biology, University of Turku, Turku, Finland; 6 Department of Horticulture, College of Agriculture and Environmental Sciences, Bahir Dar University, Bahir Dar, Ethiopia

**Keywords:** 16S rRNA gene, bitter white lupine, *Lupinus albus*, microbial communities, rhizosphere soil, soil health, triticale

## Abstract

This study aimed to elucidate the composition and diversity of microbial communities associated with bitter white lupine (BWL) root nodules, BWL rhizosphere soil, and neighboring triticale (×*Triticosecale Wittmack*) rhizosphere soil via 16S rRNA gene sequencing. Significant differences in microbial composition and diversity were observed among the sample types. BWL nodules harbored distinct bacterial communities dominated by nitrogen-fixing Bradyrhizobium (61.09%). While both BWL and triticale rhizosphere soils had high bacterial diversity dominated by Actinobacteriota and Proteobacteria (32.81 and 25.18% in BWL, and 27.73 and 26.41% in triticale, respectively). Although BWL and triticale rhizosphere soils shared some microbial taxa, each had substantial unique bacterial communities. Alpha diversity analysis revealed higher bacterial diversity in rhizosphere soils than in nodules. Edaphic factors, such as the organic carbon to clay ratio (soil health indicator), available phosphorus, and clay content, are important factors of rhizosphere microbial community structure. Positive correlations were found between soil organic matter, total nitrogen, and microbial diversity in rhizosphere soils. These findings provide novel insights into plant–microbe interactions in acidic, nutrient-poor soils of northwestern Ethiopia and suggest that microbiome management could enhance soil health and crop productivity in marginal agricultural lands.

## 1 Introduction

Bitter white lupine (BWL; *Lupinus albus* L.) is a neglected pulse crop in Ethiopia, often considered a poor man’s crop that grows in the acidic, marginal lands of the Amhara region in the northwestern part of the country. Despite its potential to improve soil fertility and provide high-protein food, limited research has focused on the microbial communities associated with BWL, particularly in the acidic, nutrient-poor soils of this region. Understanding these microbial communities is critical because plant-associated microbiomes significantly influence nutrient cycling, soil health, and crop productivity under challenging environmental conditions. While previous studies have examined rhizosphere microbiomes of major cereal such as wheat and legume crops such as faba bean, chickpea, and haricot bean, microbial assemblages specific to BWL nodules and rhizosphere soils remain understudied, limiting our ability to harness beneficial microbes for sustainable agriculture in these marginal lands. Investigating the microbial communities of BWL and its neighboring cereal crop, triticale, in this understudied region addresses a significant knowledge gap and offers insights into plant–microbe interactions that could enhance soil fertility and crop resilience.

BWL is characterized by high protein (50.97%) and alkaloid (1.43%) contents (Yeheyis et al., 2011; Kefale et al., 2022). Like other leguminous crops, BWL forms symbiotic associations with nitrogen-fixing bacteria to fulfill its nitrogen requirements. Similarly, farmers commonly cultivate triticale (×*Triticosecale Wittmack*) in acidic and nutrient-poor soils, where the production of other acid-sensitive cereals is not feasible. Triticale’s ability to thrive in acidic soils can also be linked to its associated rhizosphere microbial communities.

As demonstrated by various researchers (Simmons et al., 2018; Kui et al., 2021), soil plant-associated microbiomes consist of dynamic and complex microbial communities. These microbial communities play crucial roles in agricultural ecosystems, influencing soil health (defined as the capacity of soil to function as a vital living ecosystem that sustains plants, animals, and humans), nutrient cycling, and plant productivity (Sharma et al., 2011; Alawiye and Babalola, 2019) through the production of phytohormones and the suppression of disease-causing microorganisms (Sharma et al., 2020; Das et al., 2022). Understanding the composition and diversity of these microbial communities is essential for developing sustainable farming practices that take advantage of beneficial microorganisms. However, the relationships among plant species, their associated microbiomes, and soil properties remain understudied, particularly in acidic soils and nutrient-poor environments of Ethiopia, where food production faces significant challenges.

Plant-associated microbiomes play a crucial role in nutrient cycling and soil health; however, the microbial community dynamics within BWL root nodules, its rhizosphere soil, and the rhizosphere soil of neighboring triticale remain poorly understood, especially in acidic, nutrient-poor soils of northwestern Ethiopia. We hypothesize that the microbial community composition and diversity differ significantly among BWL nodules, BWL rhizosphere soil, and triticale rhizosphere soil, reflecting distinct selective pressures from plant species and soil physicochemical properties. Specifically, we expect beneficial taxa such as *Bradyrhizobium* to be enriched within BWL nodules. This enrichment is anticipated to enhance nitrogen fixation and promote soil health and fertility in these marginal environments. To clarify these differences, this study employs 16S rRNA gene sequencing to provide insights into plant–microbe interactions that support sustainable crop productivity in challenging soils.

This study aims to elucidate the composition and diversity of microbial communities within the nodules and rhizosphere soils of BWL and compare them with those of neighboring triticale crops in northwestern Ethiopia. By characterizing these microbial communities, the study seeks to provide insights into their potential roles in enhancing soil health, fertility, and productivity in acidic and marginal lands. This comparative analysis of microbial communities associated with a legume (BWL) and a cereal (triticale) crop is regionally novel for acidic soils of northwestern Ethiopia, offering a unique opportunity to understand the complex plant–soil–microbe interactions that shape sustainable agricultural systems. However, it is important to note that differences in fertilization and tillage practices between BWL and triticale fields represent confounding factors that may influence microbial communities and limit causal inference regarding plant species effects.

## 2 Materials and methods

### 2.1 Sampling sites, rhizosphere soil, and nodule sample collection

**Figure 1:**
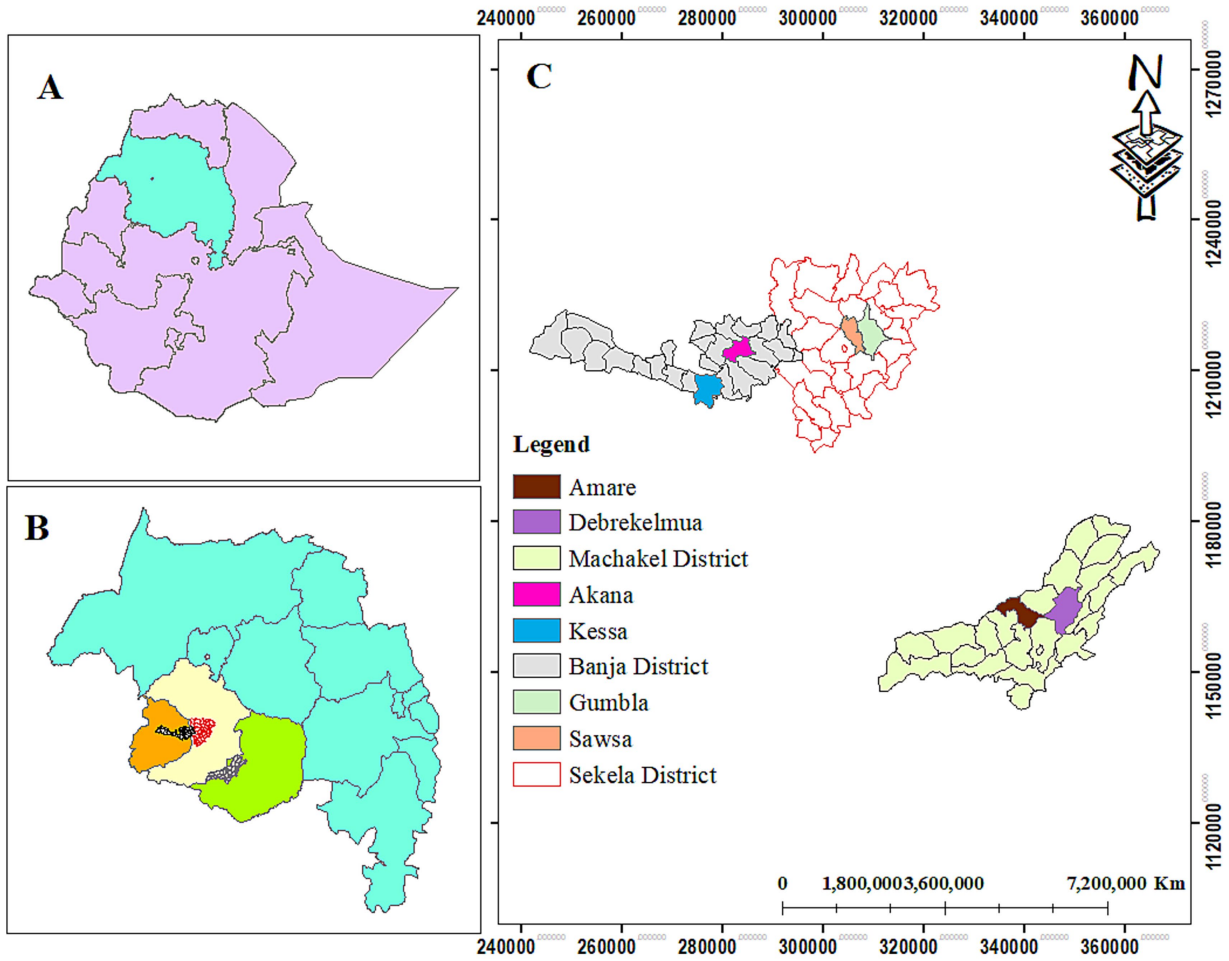
Location map of the study area in northwestern Ethiopia. Panel **(A)** shows the Amhara region within Ethiopia, Panel **(B)** highlights the three study districts (Machakel, Banja, and Sekela), and Panel **(C)** identifies the six specific study sites (Akana, Kessa, Amare, Debrekelmua, Gumbla, and Sawsa) within these districts.

Rhizosphere soil samples (36 in total) were collected from BWL and neighboring triticale fields in northwestern Ethiopia across six sites, including Akana, Kessa, Amare, Debrekelmua, Gumbla, and Sawsa (Figure 1). At each site, three separate BWL fields and three separate neighboring triticale fields were sampled, yielding six replicates per site (three per crop). This design resulted in 18 BWL rhizosphere samples and 18 triticale rhizosphere samples. These selected sites are known for BWL production in the northwestern part of Ethiopia across three districts (Machakel, Banja, and Sekela). The neighboring fields for each crop were selected based on similar management practices, specifically cropping history/crop rotation and expected soil homogeneity to minimize confounding factors. However, fertilization regimes differed between crops, with triticale fields receiving fertilizer applications while BWL fields did not. Tillage practices also differed (BWL was cultivated with a single tillage operation at sowing, whereas triticale underwent three to four tillage operations). These differences may influence soil physicochemical properties and rhizosphere microbial communities, limiting the ability to attribute observed microbial differences solely to plant species effects. These differences are acknowledged as limitations of the study design.

Rhizosphere soil samples were collected from each BWL at the flowering-to-pod-setting stage and from triticale crops at the flowering stage to the grain-filling period. At each site, four to seven plants per replicate field were randomly selected, and their rhizosphere soil was pooled to represent one composite sample. This compositing approach was chosen to capture average field-level microbial communities while optimizing sequencing resources. However, compositing reduces resolution of plant-to-plant rhizosphere variations and may mask intra-filed heterogeneity. Consequently, statistical comparisons are limited to field-level averages, and the results should be interpreted as representative of the field scale rather than individual plant variability. Loosely adhering soil was removed and tightly adhering soil to the roots was collected by vigorously shaking roots into sterile containers. Root nodule samples were also collected from each BWL plant across the selected sites. Several undamaged and healthy nodules were harvested per plant per site and stored in sterilized 15 mL centrifuge tubes containing silica gel (desiccant agent) following the instructions described by Howieson and Dilworth (2016). The rhizosphere soil samples and nodule tubes were stored in an icebox until they were preserved in the laboratory at −20 °C and 4 °C, respectively, for microbiome and genetic diversity studies at the University of Helsinki (UH), Finland. Some of the soil samples were air-dried in the shade for physico-chemical property analysis.

### 2.2 Soil physicochemical analysis

Soil particle distribution was analyzed by determining the percentage of sand and clay fractions in each soil sample by following the Bouyoucos hydrometer method (Bouyoucos, 1962). Soil pH was measured potentiometrically in a 1:2.5 soil-to-water suspension, following the procedure outlined by Sahlemedhin and Taye (2000). Soil organic carbon (OC) was determined by the Walkley-Black wet oxidation method (Walkley and Black, 1934), and total nitrogen (TN) content of the soil was determined by the Kjeldahl digestion and distillation method as described by Bremner (1965). Available phosphorus (AvP) content of the soil was determined using the Olsen extraction and spectrophotometry method (Olsen, 1954), and cation exchange capacity (CEC) was measured using 1 N ammonium acetate extraction, followed by displacement with a neutral salt, and quantified titrimetrically after distillation of the displaced ammonium. A soil health indicator (SHI) was calculated from the ratio of OC to clay as described by Johannes et al. (2017) and Prout et al. (2021). Accordingly, the SHI values reported in this manuscript represent the corresponding carbon-to-clay ratio. While this approach has been applied in soil health assessments, it is not yet widely standardized in microbiome studies. Therefore, in this study the OC/clay ratio is treated as one of the exploratory variables.

### 2.3 Rhizosphere soil and root nodule DNA extraction

The genetic material (DNA) was extracted from 250 mg of rhizosphere soil samples collected from BWL and triticale, as well as from BWL root nodules per plant. Extractions were performed using a NucleoSpin Soil DNA Isolation Kit following the manufacturer’s instructions (Macherey-Nagel GmbH Co. KG, Duren, Germany), as described by Mousavi et al. (2022). Before nodule DNA extraction, the nodules were surface sterilized in 96% ethanol for 1 min and in 4% NaClO for 3–5 min, followed by 4–6 repeated washes with autoclaved Milli-Q water. To verify the effectiveness of the sterilization protocol, 1–2 drops of the final rinse water were plated onto yeast extract mannitol agar (YEMA) and incubated for up to 7 days; no visible bacterial growth was detected, confirming the removal of surface contaminants. Negative controls, including DNA extraction blanks and PCR no-template controls, were included throughout the DNA extraction and amplification processes, and no DNA or PCR products were detected in these controls, indicating the absence of contamination during sample processing. In total, 36 and 54 DNA samples were isolated from the rhizosphere soil and nodule samples (nodules of three plants per replication at each site were randomly selected), respectively. The quality and quantity of the DNA samples were checked via a Thermo Scientific NanoDrop Spectrophotometer (Thermo Fisher Scientific, Waltham, Massachusetts, U.S.) and 1% agarose gel electrophoresis.

### 2.4 PCR (polymerase chain reaction) and Amplicon sequencing

The highly variable region (V3–V4) of the bacterial 16S rRNA gene was amplified from all DNA samples isolated from the rhizosphere soil and nodule samples using the primer pair Illum_341F and Illum_785R, following the methods described by Herlemann et al. (2011) and Thijs et al. (2017). The PCR preparations, reaction cycles, and polymerase enzymes were performed following the working package designed by the Finnzymes Phusion Hot Start High Fidelity DNA Polymerase (FINNZYMES OY Keilaranta, Espoo, Finland). PCRs were carried out with a 25 μL reaction mixture of Phusion Hot-start High-Fidelity PCR Master Mix, 0.5 μL of forward and reverse primers per reaction in 25 μL of PCR, and 1 μL of template DNA. Thermal cycling consisted of initial denaturation at 98 °C for 30 s, followed by 27 cycles of denaturation at 98 °C for 30 s, annealing at 55 °C for 30 s, and elongation at 72 °C for 30 s. Finally, the samples were incubated at 72 °C for 5 min. The PCR product quality was checked via 1% agarose gel electrophoresis as described above. Amplicons were sequenced via Illumina MiSeq sequencing (2 × 300 bp paired-end) at the Institute of Biotechnology Sequencing Core Facility, University of Helsinki.

### 2.5 Bioinformatics and amplicon sequence data analysis

The sequences were preprocessed using the Fastq program to remove errors and bad-quality sections, after which the primers and adapters were trimmed with *Cutadapt* (Martin, 2011). Amplicon sequence variants (ASVs) were processed via the *DADA2 (Divisive Amplicon Denoising Algorithm)* pipeline (Callahan et al., 2016). This included quality filtering, error correction, denoising, paired-end read merging, chimera removal, and taxonomic classification. Sequences were truncated at 260 bp (forward reads) and 200 bp (reverse reads) based on quality profiles. Reds were filtered with maxN = 0, truncQ = 2, and maximum expected errors (maxEE) set to 2 for forward and 5 for reverse reads. Reads shorter than 200 bp were discarded, and phiX sequences were removed. Error models were estimated using the DADA2 default algorithm. After denoising and merging paired-end reads, chimeras were removed using the consensus method. Rarefaction was performed at a depth of 17,016 sequences per sample to standardize sampling effort across samples.

After preanalysis, the resulting sequences were compared with the SILVA SSU Ref NR 99 database (release v138.1; silva_nr99_v138.1_train_set.fa) and classified into ASVs (Quast et al., 2012; I-Hsuan, 2021). This release was selected because it provides the most up-to-date, curated, and taxonomically consistent reference set available at the time of analysis, ensuring accuracy and reproducibility in taxonomic classification despite containing fewer sequences than earlier releases. Rarefaction curves were generated via the *ggrare* function from the *ranacapa* package of R (Kandlikar et al., 2018), with a standardized step size of 50, to visualize sequencing depth and species richness across samples. The curves were plotted via *ggplot2* in R (Wickham, 2016). Bacterial relative abundance at the phylum, class, and genus levels was calculated and visualized via the *phyloseq* (McMurdie and Holmes, 2013), *dplyr* (Wickham et al., 2023), and *ggplot2* (Wickham, 2016) packages in R.

The *Venn diagram* package (Chen, 2018) was used to visualize the unique and shared ASVs among different sample types. The alpha diversity indices (observed species, Shannon and Simpson diversity, and evenness) were calculated and analyzed in the R program using the *phyloseq* (McMurdie and Holmes, 2013), *microbiome* (Lahti et al., 2017), and *FSA (Fisheries Stock Analysis*; Ogle et al., 2023) packages. The beta diversity of microbial communities across samples was assessed using Principal Coordinate Analysis (PCoA) based on Bray–Curtis dissimilarities.

### 2.6 Statistical analyses

Differences in soil properties were analyzed using two-way ANOVA in R (R Core Team, 2024), followed by a *post hoc* Tukey’s honestly significant difference (HSD) test (Tukey, 1949). Microbial alpha diversity indices (observed ASVs, Shannon, and Simpson indices) were analyzed using Aligned Rank Transform (ART) ANOVA to account for nonnormality and interaction effects between sample type and sampling site. *Post hoc* comparisons used Tukey’s HSD for main effects and Benjamini–Hochberg correction for interaction terms.

Two-way PERMANOVA was conducted via the *adonis2* function from the *vegan* package (Oksanen et al., 2013) with 999 permutations to assess the effects of sample type, sampling site, and their interaction on community structure. The homogeneity of multivariate dispersions was tested using PERMDISP (betadisper function, vegan package) with 999 permutations to verify a key assumption of PERMANOVA. Significant results were followed by pairwise comparisons *via pairwise Adonis*, with Bonferroni-adjusted *p*-values for multiple testing.

The PCoA was conducted via the *cmdscale* function from the *vegan* package (Oksanen et al., 2013), and the variance explained by the principal coordinates was calculated. To assess correlations, the soil parameters were overlaid as vectors via *envfit (vegan)* with 999 permutations. The dbRDA (distance-based redundancy analysis) was performed via the *capscale* function, where the Bray–Curtis distance matrix was modeled against selected environmental variables. A forward selection procedure was applied to identify the most significant soil variables influencing community composition via the *ordiR2step* function (Oksanen et al., 2013). Differential abundance was tested via *DESeq2* (Love et al., 2014) and visualized with volcano plots generated using *ggplot2*. While *DESeq2* does not explicitly account for the compositional nature of microbiome data, recent benchmarks report false-positive rates below 15% under realistic conditions (Agudelo and Miller, 2025). To further mitigate potential bias, low-abundance taxa (total counts < 10 across all samples) were filtered and a stringent false discovery rate (FDR) correction (adj. *p* < 0.1) was applied.

Differentially abundant microbial taxa were identified based on log2-fold-change (FC) values and adjusted *p* values (adj. *p* < 0.1). The enriched taxa were classified as significantly more abundant in BWL rhizosphere soil, triticale rhizosphere soil, or BWL nodules. Correlation analyses were conducted to explore the relationships between alpha diversity metrics (observed, Shannon, and Simpson indices) and selected soil physicochemical properties. Pearson’s correlation coefficients were calculated using a custom R function *(cor.test())* from base R (Benesty et al., 2009).

## 3 Results

### 3.1 Physicochemical characteristics of the soil samples

The analysis of rhizosphere soil properties revealed significant variations across sampling sites, whereas differences between BWL and triticale rhizosphere soils were generally not statistically significant (Table 1, Supplementary Table 1).

**Table 1:** Physicochemical properties of rhizosphere soils across sampling sites and sample types.

Sampling__site	Sample_type	Soil parameters								
		pH (1:2.5 H_2_O)	OM (%)	TN (%)	C:N (ratio)	AvP (mg/kg)	CEC (meq/100 g)	Sand (%)	Clay (%)	SHI (OC to clay ratio)
Akana	BWL_RS	5.04 ± 0.69ab	3.70 ± 1.47b	0.15 ± 0.03b	13.73 ± 2.98a	20.39 ± 13.46a	9.87 ± 1.50b	49.67 ± 5.51bc	34.33 ± 8.02b	0.068 ± 0.039bc
Akana	Triticale_RS	5.57 ± 0.74ab	3.75 ± 2.45b	0.16 ± 0.06b	12.56 ± 5.60a	28.10 ± 25.56a	12.07 ± 3.32b	52.67 ± 9.29bc	32.67 ± 13.61b	0.086 ± 0.073bc
Kessa	BWL_RS	5.06 ± 0.09b	7.17 ± 1.12a	0.25 ± 0.05a	16.95 ± 1.03a	6.35 ± 4.50b	26.27 ± 9.06a	60.67 ± 3.06a	22.00 ± 4.00c	0.197 ± 0.067a
Kessa	Triticale_RS	5.03 ± 0.09b	8.12 ± 0.10a	0.32 ± 0.05a	15.06 ± 2.20a	6.58 ± 5.34b	14.67 ± 2.05a	57.67 ± 1.53a	20.00 ± 00c	0.236 ± 0.003a
Amare	BWL_RS	5.19 ± 0.05ab	4.15 ± 0.51b	0.17 ± 0.03b	14.50 ± 2.27a	9.69 ± 4.65ab	14.80 ± 2.20ab	47.67 ± 2.52c	37.00 ± 3.61b	0.066 ± 0.012bc
Amare	Triticale_RS	5.34 ± 0.03ab	3.81 ± 0.98b	0.16 ± 0.05b	13.58 ± 0.30a	17.60 ± 9.37ab	11.87 ± 0.46ab	47.00 ± 5.29c	38.67 ± 8.33b	0.061 ± 0.026bc
Debrekelmua	BWL_RS	5.03 ± 0.08b	1.34 ± 0.61c	0.10 ± 0.01b	7.85 ± 2.75b	13.97 ± 9.01ab	11.13 ± 0.61b	41.33 ± 2.52d	46.00 ± 5.29a	0.018 ± 0.010c
Debrekelmua	Triticale_RS	5.24 ± 0.20b	1.68 ± 0.58c	0.11 ± 0.04b	8.72 ± 0.99b	12.37 ± 3.76ab	13.40 ± 0.87b	38.00 ± 2.00d	52.33 ± 3.51a	0.019 ± 0.007c
Gumbla	BWL_RS	5.71 ± 0.11a	2.75 ± 0.26bc	0.14 ± 0.01b	11.19 ± 0.67a	8.98 ± 1.87ab	11.33 ± 2.41b	51.00 ± 1.00c	33.33 ± 4.16b	0.048 ± 0.004bc
Gumbla	Triticale_RS	5.77 ± 0.05a	2.69 ± 0.58bc	0.11 ± 0.02b	14.47 ± 0.47a	21.61 ± 7.81ab	11.00 ± 1.40b	48.00 ± 6.24c	35.17 ± 10.13b	0.048 ± 0.021bc
Sawsa	BWL_RS	4.91 ± 0.13b	3.92 ± 1.52b	0.14 ± 0.02b	15.88 ± 4.08a	11.86 ± 6.31ab	12.87 ± 4.61b	54.67 ± 1.53ab	29.33 ± 3.21bc	0.080 ± 0.036b
Sawsa	Triticale_RS	5.27 ± 0.13b	5.49 ± 1.72b	0.19 ± 0.04b	16.81 ± 1.96a	21.69 ± 9.20ab	11.8 ± 5.57b	59.67 ± 2.52ab	26.00 ± 5.29bc	0.131 ± 0.059b

Means followed by the same letter within a soil property are not significantly different according to Tukey’s HSD test (*p* < 0.05). BWL_RS = bitter white lupine rhizosphere soil; Triticale_RS = triticale rhizosphere soil; OM = organic matter; TN = total nitrogen; C:N = carbon-to-nitrogen ratio; AvP = available phosphorus; CEC = cation exchange capacity; SHI = soil health indicator (OC/clay). The lowercase letters (a, b, c, d, e) denote statistical groupings based on post hoc multiple comparisons (Tukey’s HSD for soil properties).

The soil pH ranged from 4.91 to 5.77 across all the sites and sample types, with significant differences between the sampling sites (*p* = 0.008). A slight but statistically significant difference in pH was observed between the BWL and triticale rhizospheres (*p* = 0.05), with the triticale rhizosphere maintaining a marginally higher pH. The organic matter (OM) content varied significantly across the sampling sites (*p* < 0.001), with Kessa exhibiting the highest mean values for both triticale (8.12%) and BWL (7.17%) rhizospheres. The TN level differed significantly among the sites (*p* < 0.001) but not between the two crop rhizospheres (*p* = 0.192). The carbon to nitrogen (C:N) ratio also showed significant site-specific differences (*p* < 0.001), with the lowest values recorded at Debrekelmua for both crop types.

Available phosphorus levels did not differ significantly between rhizosphere types (*p* = 0.088) or across sampling sites (*p* = 0.136), indicating relatively uniform phosphorus availability throughout the study area. The CEC varied significantly across sites (*p* = 0.002) but not between crop types. The soil texture components (sand, clay, and silt) also presented significant site-specific differences (*p* < 0.001 to <0.01). The SHI, calculated as the ratio of OC content to clay content, differed significantly among sites (*p* < 0.001) but not among rhizosphere types (*p* = 0.752). Kessa exhibited the highest SHI values for both the triticale (0.236) and BWL (0.197) rhizospheres, suggesting better soil stability and OM retention at this site.

### 3.2 Microbial community composition and distribution in BWL nodules and rhizosphere soils

Illumina MiSeq sequencing of 90 samples (36 rhizosphere soil and 54 nodule samples) produced 9,530,060 raw reads, with individual counts ranging from 70,462 to 156,122. After quality filtering and chimera removal, 4,393,867 high-quality sequences remained, yielding 60,740 ASV occurrences across all the samples (Supplementary Table 2). The total ASV count may reflect fine-scale sequence variants resolved by the DADA2 algorithm. Stringent filtering criteria, including chimera removal and quality thresholds, were applied to minimize spurious ASVs. Nonetheless, this high ASV number suggests considerable microbial diversity across the rhizosphere and nodule samples. Rarefaction curves were generated at a step size of 75 reads and indicated that the sequencing depth was sufficient to represent the bacterial communities in all the samples (Supplementary Figure 1). To ensure transparency and reproducibility, a comprehensive supplementary table (Supplementary Table 4) is provided, which detailing all detected ASVs together with their taxonomic assignments and sample-specific distributions.

**Figure 2:**
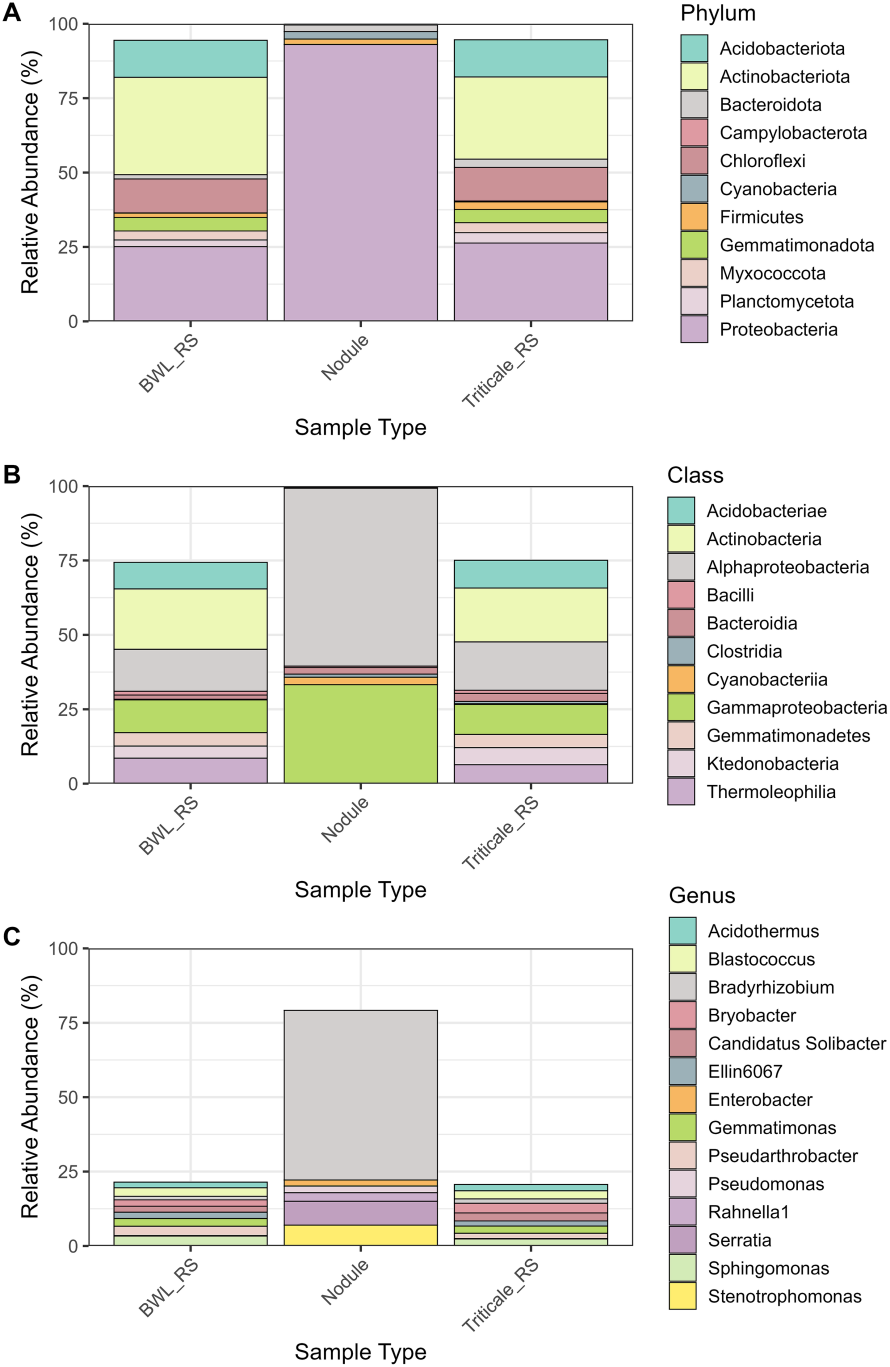
Relative abundance of dominant microbial taxa at the **(A)** phylum, **(B)** class, and **(C)** genus levels across sample types: bitter white lupine rhizosphere soil (BWL_RS), triticale rhizosphere soil (Triticale_RS), and BWL root nodules (Nodule), showing the union of the six most abundant taxa per group; legends may therefore include more than six taxa.

High-throughput sequencing identified 40 phyla, 97 classes, 311 families, and 773 genera. Figure 2 presents the relative abundances of the top six dominant microbial taxa at the phylum, class, and genus levels for each sample type. In BWL nodules, Proteobacteria dominated (93.07%), whereas Bacteroidota and Firmicutes accounted for 2.20 and 1.85%, respectively. At the genus level, *Bradyrhizobium* was more abundant in BWL nodules (61.09%) than in BWL rhizosphere soil (2.11%) and triticale rhizosphere soil (2.55%). Other notable genera in BWL nodules included *Serratia*, *Stenotrophomonas*, *Rahnella*, *Pseudomonas*, and *Enterobacter*. In contrast, both the BWL and triticale rhizosphere soils were dominated by Actinobacteriota (32.81% in BWL rhizosphere soil vs. 27.73% in triticale rhizosphere soil) and Proteobacteria (25.18% in BWL rhizosphere soil vs. 26.41% in triticale rhizosphere soil), with additional phyla such as Acidobacteriota, Chloroflexi, and Gemmatimonadota present in both rhizosphere soils (Figure 2).

**Figure 3:**
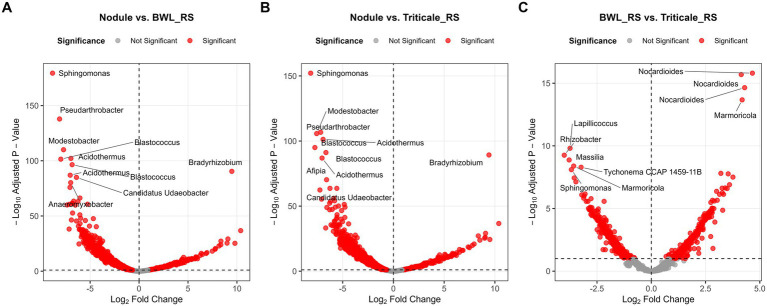
Volcano plots depicting the differential abundance of microbial taxa at the genus level for **(A)** nodule vs. BWL_RS, **(B)** nodule vs. Triticale_RS, and **(C)** BWL_RS vs. Triticale_RS. Red points denote taxa with significant abundance differences (adj. *p* < 0.1). Positive log2FC values indicate enrichment in the first group of each comparison. Labeled taxa represent the top 10 most significant genera.

Differential abundance analysis at the genus level revealed significant differences among the sample types (Figure 3). The BWL rhizosphere soil was significantly enriched in soil-associated taxa such as *Nocardioides* [log2-fold change (log2FC) > 4.10, adj. *p* < 2.05E-16] and *Marmoricola* (log2FC of 4.19, adj. *p* = 2.12E-14). In contrast, the genus *Tychonema CCAP 1459-11B* was significantly depleted in the BWL rhizosphere soil (log2FC = −3.23, adj. *p* = 5.09E-09).

Furthermore, *Bradyrhizobium* was highly enriched in the BWL nodules, with a log2FC of 9.48 (adj. *p* = 3.49E-91) compared with BWL rhizosphere soil and 9.42 (adj. *p* = 5.17E-90) compared with triticale rhizosphere soil. The analysis also revealed a marked depletion of *Acidothermus* in the nodule samples, with log2FC values ranging from −7.04 to −6.96 compared with those in BWL rhizosphere soil and from −7.03 to −6.93 (adj. *p* = 1.21E-87 to 5.15E-102) compared with triticale rhizosphere soil. Moreover, the genus *Sphingomonas* was significantly depleted in the nodules compared with both the BWL and triticale rhizosphere soil samples, with log2FC values of −8.84 (adj. *p* = 4.01E-180) and −8.16 (adj. *p* = 7.24E-153), respectively. Key differences in microbial taxon abundance across sample types are visualized in volcano plots (Figures 3A–C).

**Figure 4:**
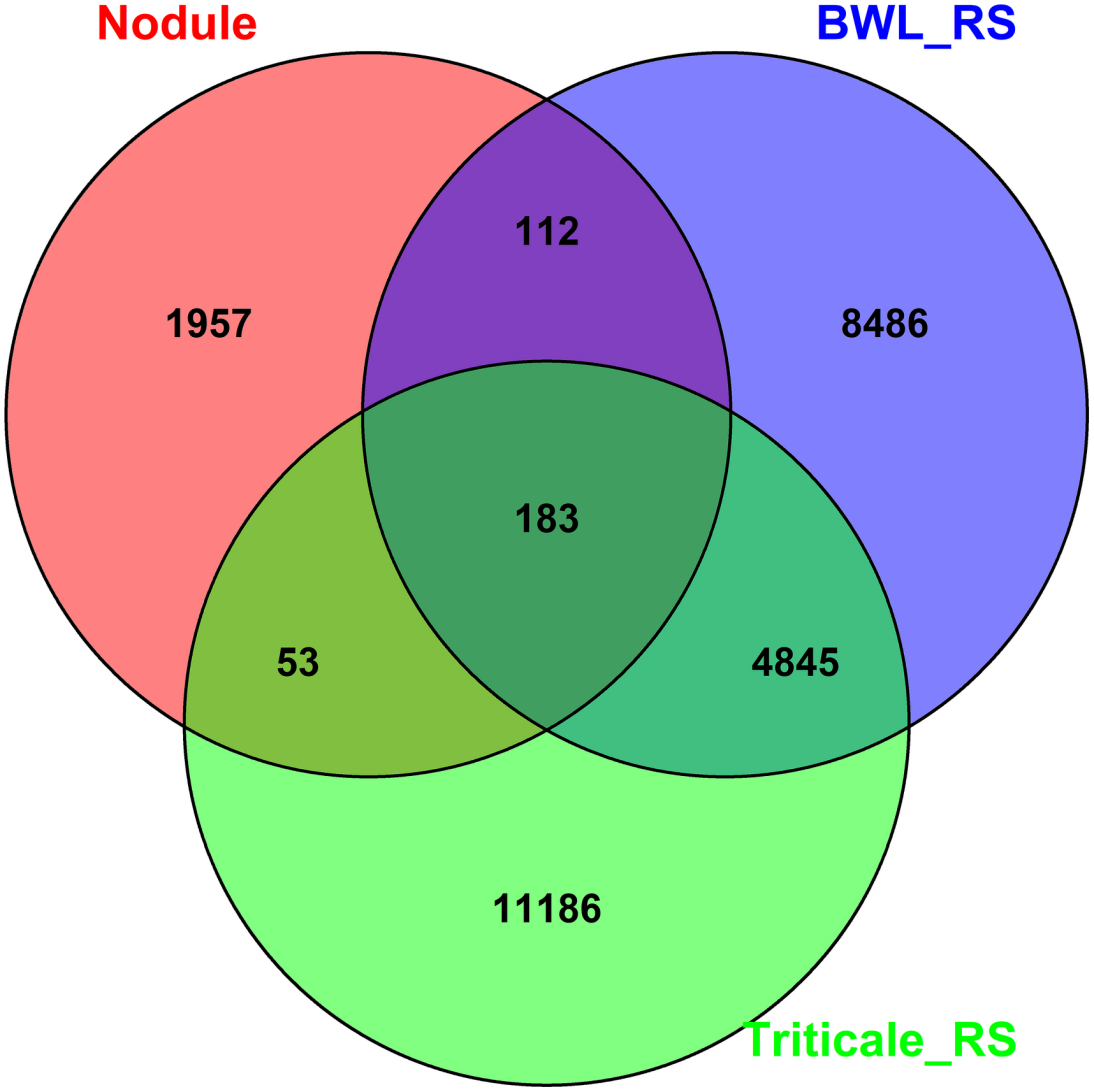
Venn diagram showing the number of shared and unique ASVs among BWL root nodules, BWL rhizosphere soil (BWL_RS), and triticale rhizosphere soil (Triticale_RS).

The Venn diagram (Figure 4) illustrates the distribution and overlap of amplicon sequence variants (ASVs) among BWL rhizosphere soil, triticale rhizosphere soil, and BWL nodules. The analysis revealed that the BWL and triticale rhizosphere soil samples harbored 8,486 and 11,186 unique ASVs, respectively, and shared 5,028 ASVs. In contrast, the BWL nodules presented a unique composition with 1,957 ASVs and shared 295 ASVs with the BWL rhizosphere soil and 236 ASVs with the triticale rhizosphere soil. Notably, only 183 ASVs were common to all three sample types, representing a small core microbiome common to both plant species and the nodule environment.

### 3.3 Alpha diversity of microbial communities

The Aligned Rank Transform ANOVA (ART ANOVA; Supplementary Table 3) revealed significant variation among sample types across all diversity indices (observed: *p* < 0.001, *η*^2^*p* = 73.16%; Shannon: *p* < 0.001, *η*^2^*p* = 74.73%; Simpson: *p* < 0.001, *η*^2^*p* = 65.11%). Although the microbial diversity of the two rhizosphere soils was not statistically different, the triticale rhizosphere exhibited a greater diversity compared to the BWL rhizosphere. In contrast, the nodules showed lower microbial diversity relative to both rhizosphere soils (Table 2). A significant interaction effect between sample type and sampling site was observed solely for the observed ASVs (*p* < 0.001). Notable site-specific differences in the Simpson index were particularly evident between Debrekelmua and Akana.

**Table 2:** Mean (±standard deviation) observed ASV, Shannon, and Simpson diversity indices for sample types across sampling sites.

Sampling site	Sample type	Observed ASVs (mean ± SD)	Shannon index (mean ± SD)	Simpson index (mean ± SD)
Akana	Triticale_RS	1,732 ± 193.08a	6.88 ± 0.18a	0.998 ± 0.001a (a)
Amare		1,600 ± 414.65a	6.58 ± 0.38a	0.995 ± 0.005a (ab)
Debrekelmua		1,284 ± 134.22ade	6.57 ± 0.09a	0.998 ± 0.000a (b)
Gumbla		1,438 ± 256.51a	6.67 ± 0.13a	0.998 ± 0.000a (ab)
Kessa		2028 ± 230.66a	7.01 ± 0.08a	0.998 ± 0.000a (ab)
Sawsa		1,579 ± 49.80a	6.74 ± 0.10a	0.998 ± 0.000a (ab)
Akana	BWL_RS	1,630 ± 189.95a	6.59 ± 0.02b	0.997 ± 0.001a (a)
Amare		1,435 ± 165.62a	6.52 ± 0.13b	0.997 ± 0.001a (ab)
Debrekelmua		1,264 ± 113.18abde	6.42 ± 0.02b	0.996 ± 0.001a (b)
Gumbla		1,502 ± 131.25a	6.61 ± 0.13b	0.997 ± 0.001a (ab)
Kessa		1,563 ± 276.24a	6.70 ± 0.23b	0.997 ± 0.001a (ab)
Sawsa		1,290 ± 257.23ae	6.16 ± 0.70b	0.987 ± 0.018a (ab)
Akana	Nodule	103 ± 40.69bc	2.08 ± 0.95c	0.692 ± 0.263b (a)
Amare		114 ± 49.93bcd	1.61 ± 0.83c	0.535 ± 0.268b (ab)
Debrekelmua		98 ± 48.03c	1.26 ± 1.07c	0.428 ± 0.340b (b)
Gumbla		101 ± 71.22c	1.43 ± 1.19c	0.459 ± 0.303b (ab)
Kessa		101 ± 63.56bc	1.92 ± 1.32c	0.584 ± 0.379b (ab)
Sawsa		117 ± 35.51bcde	1.86 ± 0.38c	0.645 ± 0.117b (ab)

Lowercase letters denote statistical groupings based on post hoc comparisons (ART ANOVA with Tukey’s HSD; *α* = 0.05). Groups that shared at least one letter indicate no significant difference (*p* ≥ 0.05); different letters denote significant differences (*p* < 0.05). For the Simpson index, letters denote sample type groupings, while parenthetical letters reflect site-specific differences. BWL_RS = bitter white lupine rhizosphere soil; Triticale_RS = triticale rhizosphere soil; Nodule = bitter white lupine root nodules. The lowercase letters (a, b, c, d, e) denote statistical groupings based on post hoc multiple comparisons ART ANOVA with Tukey’s HSD for diversity indices.

Pairwise comparisons specific to the sampling sites revealed significant differences in the observed ASVs among the sample types across all the sites (Table 3). Nodule samples consistently demonstrated the lowest diversity in comparison with both BWL and triticale rhizosphere soils. BWL and triticale rhizosphere soils did not significantly differ in the observed ASVs at any site (adj. *p* > 0.25), indicating comparable microbial richness within these rhizosphere soils.

**Table 3:** Sampling site-specific pairwise comparisons of observed ASVs between sample types via Dunn’s test with Benjamini–Hochberg adjustment.

Sampling site	Comparison	*Z* value	Adj. *p* value
Kessa	BWL_RS – Nodule	2.12	0.025*
	BWL_RS – Triticale_RS	−0.64	0.261
	Nodule – Triticale_RS	−2.91	0.005**
Akana	BWL_RS – Nodule	2.24	0.019*
	BWL_RS – Triticale_RS	−0.46	0.324
	Nodule – Triticale_RS	−2.80	0.008**
Amare	BWL_RS – Nodule	2.47	0.010*
	BWL_RS – Triticale_RS	−0.09	0.464
	Nodule – Triticale_RS	−2.58	0.015*
Debrekelmua	BWL_RS – Nodule	2.46	0.010*
	BWL_RS – Triticale_RS	−0.09	0.464
	Nodule – Triticale_RS	−2.57	0.015*
Gumbla	BWL_RS – Nodule	2.69	0.011*
	BWL_RS – Triticale_RS	0.27	0.392
	Nodule – Triticale_RS	−2.35	0.014*
Sawsa	BWL_RS – Nodule	2.12	0.025*
	BWL_RS – Triticale_RS	−0.64	0.261
	Nodule – Triticale_RS	−2.91	0.005**

BWL_RS = bitter white lupine rhizosphere soil; Triticale_RS = triticale rhizosphere soil; Nodule = bitter white lupine root nodules. *Z*-value = standardized test statistic from Dunn’s post-hoc test, represents the standardized difference in mean ranks between groups. Positive values indicate the first group in the comparison has higher ranks than the second. The symbols indicate levels of statistical significance and meaning that: *p < 0.05, **p < 0.01.

### 3.4 Associations between microbial diversity, community composition, and environmental factors

Correlation analysis presented in Table 4 explores the relationships between bacterial alpha diversity and selected soil physicochemical properties across BWL and triticale rhizosphere soil samples. In triticale rhizosphere soil, OM showed a significant positive correlation with observed ASV richness (*r* = 0.590, *p* < 0.05) and Shannon diversity (*r* = 0.528, *p* < 0.05), suggesting an association between OM levels and microbial richness and evenness. Similarly, the SHI exhibited strong positive correlations with observed (*r* = 0.599, *p* < 0.05) and Shannon (*r* = 0.579, *p* < 0.05) indices, showing that higher OC relative to clay content was statistically associated with more diverse microbial communities, though this indicator is not yet standardized in microbiome studies. TN was also positively correlated with observed ASV richness (*r* = 0.547, *p* < 0.05), highlighting a positive statistical association between nitrogen availability and microbial diversity in triticale rhizospheres. In contrast, no statistically significant correlations were found in the BWL rhizosphere soil, although weak positive trends for the OM, TN, and SHI were observed, suggesting that crop specific differences may influence how microbial diversity responses to soil properties.

**Table 4:** Correlation analysis between bacterial alpha diversity and soil physicochemical properties.

Soil parameters	BWL_RS			Triticale_RS		
	Observed	Shannon	Simpson	Observed	Shannon	Simpson
pH	−0.033	0.164	0.172	−0.161	−0.070	0.015
OM	0.261	0.090	−0.136	0.590*	0.528*	0.096
TN	0.418	0.290	0.069	0.547*	0.459	0.055
C:N	0.017	−0.225	−0.403	0.434	0.417	0.087
AvP	−0.123	0.068	0.160	−0.138	−0.143	−0.215
CEC	−0.060	0.023	−0.045	0.198	0.200	0.114
Sand	0.236	0.122	−0.100	0.418	0.421	0.100
Clay	−0.259	−0.128	0.102	−0.441	−0.451	−0.109
Silt	0.244	0.108	−0.078	0.400	0.421	0.106
SHI	0.328	0.186	−0.065	0.599*	0.579*	0.181

“*” indicates significant correlations at the 0.05 level. AvP = available phosphorous; OM = organic matter; TN = total nitrogen; and C:N = carbon-to-nitrogen ratio, CEC = cation exchange capacity, SHI = soil health indicator (OC/clay). BWL_RS = bitter white lupine rhizosphere soil; Triticale_RS = Triticale rhizosphere soil.

PERMDISP confirmed that multivariate dispersions did not differ significantly among sample types (*F* = 0.357, *p* = 0.723). The two-way PERMANOVA results (Table 5) revealed that the differences in microbial community composition were statistically significant across both the sampling sites and sample types. The analysis revealed that sample type explained the largest proportion of variance (28.2%, *p* < 0.001), reflecting distinct microbial assemblages in nodules versus rhizosphere soils. Whereas sampling site accounted for 7.7% of variance (*p* < 0.003), indicating environmental heterogeneity across locations. The interaction effect between sample type and sampling site accounted for 11.2% of the variance (*p* = 0.003) in the microbial community composition. This suggests that site specific factors modulate the microbial communities differently across sample types. Significant differences (adj. *p* < 0.05) were observed between nodules and both rhizosphere soil types at all the sampling sites. The most substantial differences were noted at Kessa and Sawsa, where the adjusted *p*-value for nodule comparisons was 0.006. In contrast, at the Amare site, the adjusted *p*-values were slightly higher, reaching up to 0.018, indicating less pronounced differences compared to the other sites.

**Table 5:** Results of two-way PERMANOVA and pairwise comparisons of microbial community composition (Bray–Curtis distances) across sample types and sampling sites.

PERMANOVA summary*					
	Df	Sum square	*R* ^2^	*F* value	*p* value
Sample_type	2	9.737	0.282	19.164	0.001
Sampling_site	5	2.650	0.077	2.086	0.003
Sample_type × Sampling_site	10	3.857	0.112	1.518	0.003
Residual	72	18.292	0.530	–	–
Total	89	34.535	1.000	–	–

Pairwise comparisons**			
Sampling site	Comparison	*p* value	Adjusted *p* value
Akana	Nodule vs. BWL_RS	0.007	0.011
	Nodule vs. Triticale_RS	0.005	0.011
	BWL_RS vs. Triticale_RS	0.300	0.300
Kessa	Nodule vs. BWL_RS	0.003	0.006
	Nodule vs. Triticale_RS	0.004	0.006
	BWL_RS vs. Triticale_RS	0.400	0.400
Amare	Nodule vs. BWL_RS	0.004	0.012
	Nodule vs. Triticale_RS	0.012	0.018
	BWL_RS vs. Triticale_RS	0.100	0.100
Debrekelmua	Nodule vs. BWL_RS	0.008	0.012
	Nodule vs. Triticale_RS	0.006	0.012
	BWL_RS vs. Triticale_RS	0.700	0.700
Gumbla	Nodule vs. BWL_RS	0.004	0.008
	Nodule vs. Triticale_RS	0.005	0.008
	BWL_RS vs. Triticale_RS	0.500	0.500
Sawsa	Nodule vs. BWL_RS	0.004	0.006
	Nodule vs. Triticale_RS	0.003	0.006
	BWL_RS vs. Triticale_RS	0.100	0.100

*The upper section reports the global effects of sample type, sampling site, and their interaction. **The lower section details pairwise differences between sample types within each site, adjusted via the Bonferroni method. BWL_RS = bitter white lupine rhizosphere soil; Triticale_RS = triticale rhizosphere soil; Nodule = bitter white lupine root nodules; *R*^2^ = proportion of variance explained by each term.

**Figure 5:**
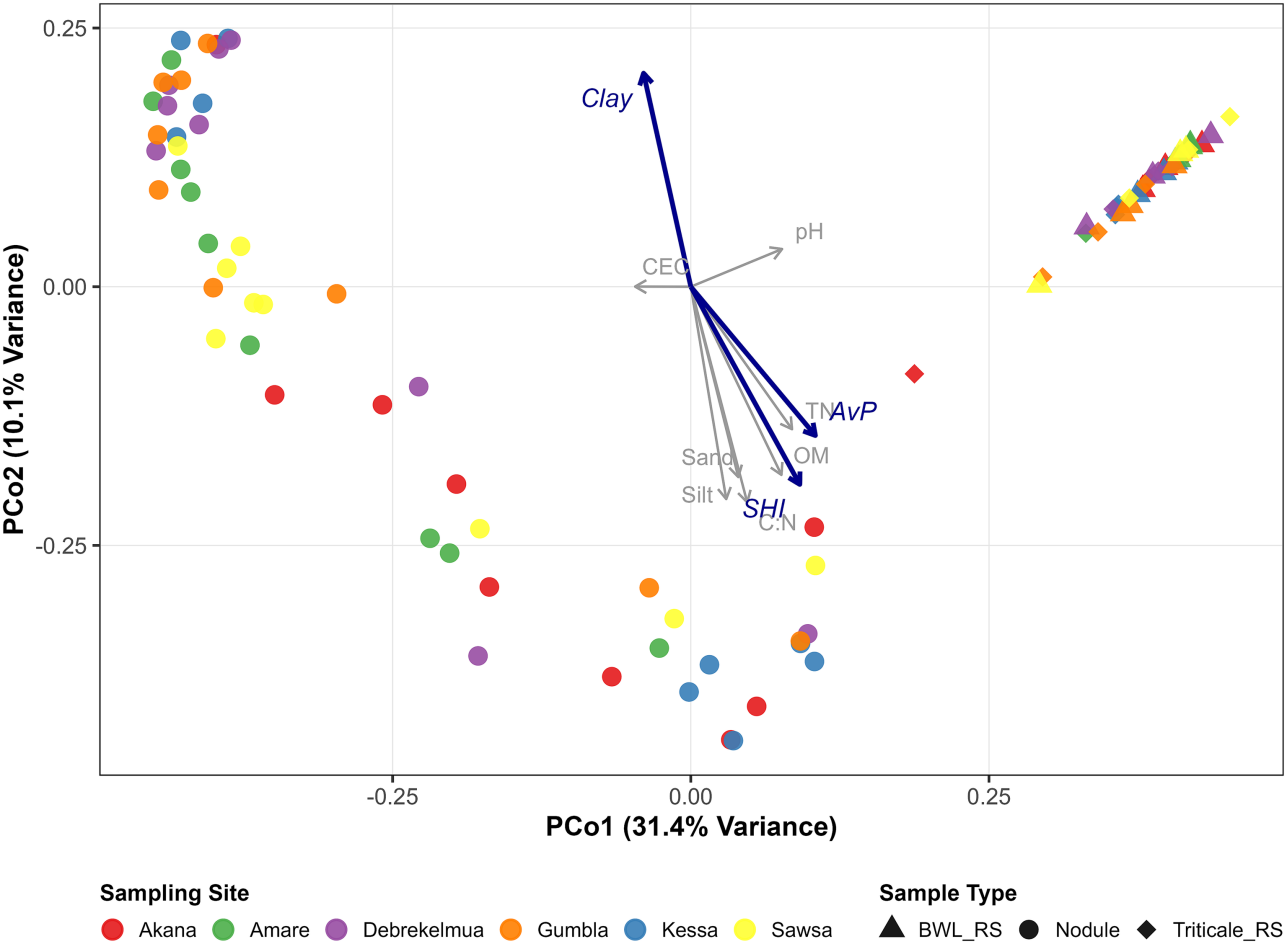
Principal coordinate analysis (PCoA) plot based on Bray–Curtis distances illustrating microbial community composition in relation to soil parameters across sampling sites and sample types. Blue arrows indicate soil parameters significantly associated with community composition. Samples are colored by sampling site and shaped by sample type.

Principal coordinate analysis (PCoA) based on Bray–Curtis distances (Figure 5) further revealed distinct clustering of samples predominantly by sample type rather than sampling site, indicating strong compositional differentiation between rhizosphere soils and nodules. The rhizosphere soil communities of BWL and triticale clustered closely, reflecting shared environmental filters.

**Figure 6:**
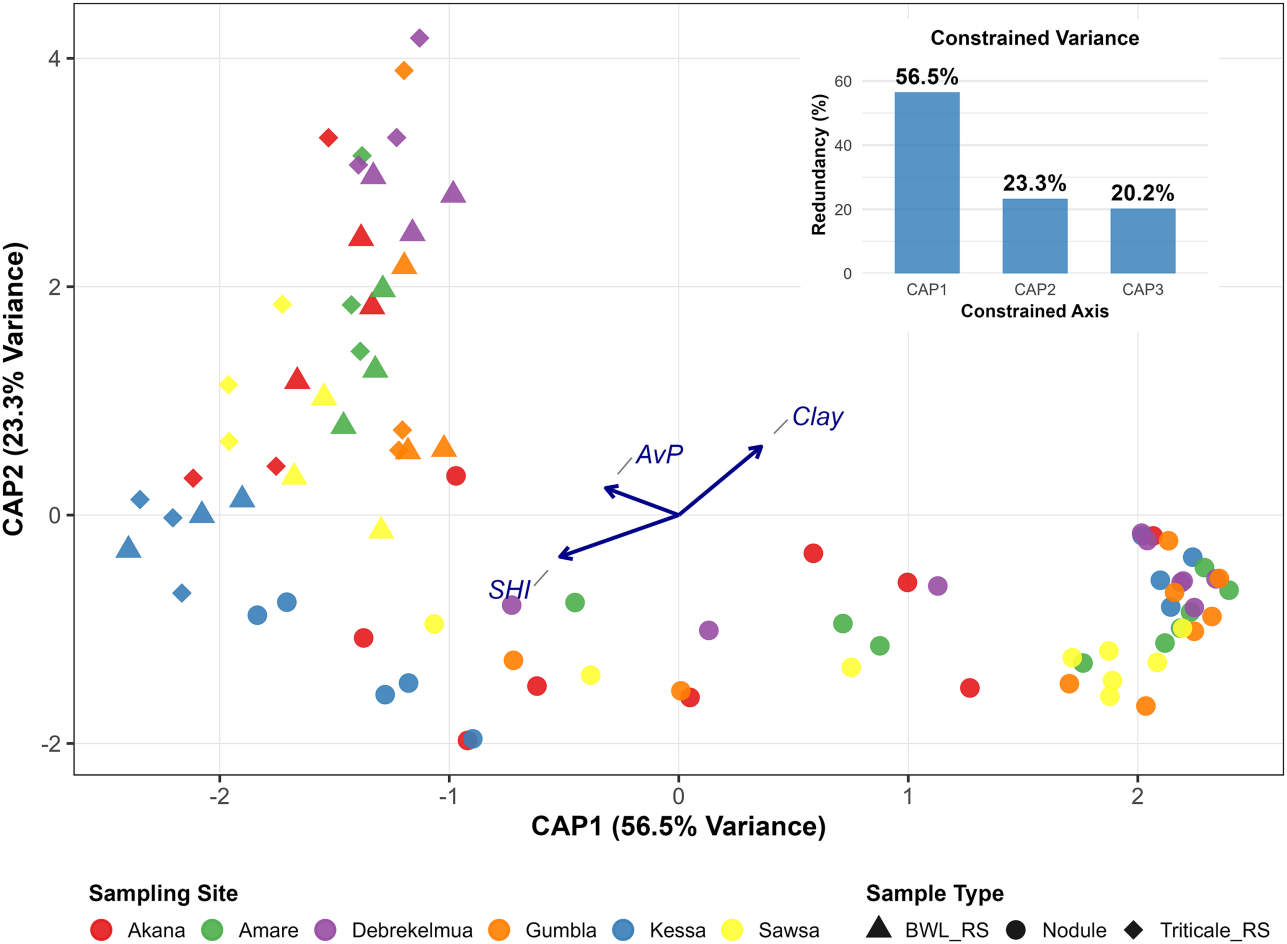
Distance-based redundancy analysis (dbRDA) of microbial community composition across sampling sites and sample types. The plot illustrates the influence of SHI (OC/clay), available phosphorus (AvP), and clay content on microbial community structure. The accompanying bar graph shows the proportion of variance explained by constrained axis (CAP1, CAP2, and CAP3).

Furthermore, distance-based redundancy analysis (dbRDA) identified SHI (*p* = 0.006), AvP (*p* = 0.016), and clay (*p* = 0.049) as explanatory environmental factors influencing community composition (Figure 6, Table 6). The constrained variance of the model was 2.813, which corresponds to 8.06% of the total variance (constrained variance), whereas the residual variance accounted for 91.94% of the variation. Among these, CAP1 accounted for 56.5% of the constrained variance (4.55% of the total variance) and separated samples primarily by sample type, which was consistent with the PCoA results, whereas CAP2 (1.88% of the total variance) revealed minor gradients linked to sampling site variability (Figure 6). Most of the nodule samples clustered at the positive end of CAP1. The BWL and triticale rhizosphere soil samples diverged along CAP2 (23.3% constrained variance), which was associated with elevated AvP and SHI.

**Table 6:** Results of distance-based redundancy analysis (dbRDA) examining the effects of environmental variables on microbial community composition.

Variable	Df	Sum square	*F*	*p* value	Variance explained (%)
SHI	1	1.075	2.88	0.006**	–
AvP	1	0.997	2.67	0.016*	–
Clay	1	0.712	1.91	0.049*	–
Constrained variance	3	2.813	–	–	8.06
Residual variance	86	32.111	–	–	91.94
Total variance	89	34.924	–	–	100

The symbols indicate levels of statistical significance and meaning that: * = *p* < 0.05, ** = *p* < 0.01.

## 4 Discussion

This study revealed significant differences in microbial composition and diversity among bitter white lupine (BWL) root nodules, BWL rhizosphere soil, and the rhizosphere soil of neighboring triticale. BWL nodules harbored distinct bacterial communities dominated by nitrogen-fixing *Bradyrhizobium*. While BWL and triticale rhizosphere soils shared some microbial compositions, each possessed substantial unique bacterial communities, with Actinobacteriota and Proteobacteria as dominant phyla. Alpha diversity analysis showed high bacterial diversity in rhizosphere soils and low diversity in nodules. Edaphic factors, including SHI (OC/clay), AvP, and clay content, were identified as important explanatory factors of rhizosphere microbial community structure. Positive associations were observed between soil OM, TN, SHI, and microbial diversity in rhizosphere soils, but SHI should be considered an informative rather than definitive soil health indicator. These findings provide insights into the potential roles of BWL nodules and rhizosphere microbiomes in promoting soil health.

### 4.1 Physicochemical characteristics of the soil samples

The significant differences in pH values among sites suggest that local environmental conditions influence soil acidity. The slight variation in pH between triticale and BWL rhizosphere soils may be due to differences in root exudation patterns. Triticale tends to maintain a slightly higher pH, potentially due to differences in rhizodeposition (the total carbon transfer from plant roots to soil) compared with BWL. Legumes like BWL release organic acids to solubilize phosphorus in nutrient-deficient soils, which acidifies the rhizosphere soil (Dakora and Phillips, 2002). Additionally, microbial community activities associated with nitrogen fixation can further influence soil acidification (Tiziani et al., 2021).

The significant variation in OM levels across sites, with the highest values occurring at Kessa, suggests differences in OM accumulation due to variations in environmental conditions or soil management practices. Higher OM at specific sites may be associated with better residue management or reduced soil disturbance. Studies have confirmed that OM levels are influenced by factors such as cropping history, residue incorporation, and climate conditions (Seddaiu et al., 2013).

The significant differences in TN levels observed across different sites suggest that nitrogen availability is influenced by factors beyond crop type. These factors might include soil microbial activity, OM decomposition, and previous land use. Although numerical differences in TN levels were noted, TN did not differ significantly between BWL and triticale rhizosphere soils. This similarity may suggest that both cropping systems contribute to nitrogen cycling; however, it is important to consider that farmers applied urea and NPS fertilizers for triticale cultivation, while no such fertilizers were used for BWL. This difference in fertilization practices could influence nitrogen availability and uptake efficiency. Research indicates that TN levels in rhizosphere soils can be affected by differences in plant nitrogen uptake efficiency (Li et al., 2008; Wang et al., 2023).

The C:N ratio, an indicator of soil OM decomposition, significantly differed across sites, with the lowest values occurring at Debrekelmua. Lower C:N ratios are often linked to more efficient nitrogen mineralization, particularly in soils with high microbial activity (Sun et al., 2021). The lower C:N values at Debrekelmua suggest higher OM decomposition rates, possibly due to microbial activity being enhanced by local soil conditions.

The lack of significant differences in the AvP levels suggests that phosphorus availability is relatively uniform across the study sites. This may be due to similar phosphorus inputs or soil properties affecting phosphorus retention and release. Soil phosphorus availability is often influenced by factors like microbial activity, which may not have varied significantly among the sites (Cai et al., 2023).

The significant differences in soil texture (sand, clay, and silt) and CEC across sites reflect the heterogeneity of soil properties among the study locations. Since CEC affects nutrient retention and availability, variations in CEC suggest that different sites have different capacities to retain and supply nutrients to plants. Soil texture differences, especially in terms of clay content, influence water-holding capacity and nutrient exchange properties, thereby affecting overall soil fertility.

The SHI differences among sites indicate variability in soil health. The highest SHI values at Kessa suggest better soil stability and OM retention. Since SHI is calculated as OC/clay, higher SHI values indicate a greater proportion of OC relative to clay, which contributes to improved soil structure and microbial activity. Higher SHI values have been associated with better soil aggregation and resilience to degradation (Sauzet et al., 2024).

### 4.2 Microbial community composition and distribution in BWL nodules and rhizosphere soils

The sequencing data revealed significant differences in microbial community composition between BWL nodules and rhizosphere soils. The dominance of Proteobacteria, particularly the high abundance of *Bradyrhizobium* (61.09%) in BWL nodules, underscores the specialized niche of root nodules in supporting symbiotic nitrogen fixation. This selective enrichment is consistent with previous studies (Sohn et al., 2021; Chaddad et al., 2024). *Bradyrhizobium* is widely reported for nitrogen fixation, and its enrichment here points to possible contributions to nitrogen availability; however, direct functional validation was not performed in this study. Moreover, while 16S rRNA gene sequencing provides broad taxonomic profiling, it has limited resolution for distinguishing closely related rhizobia species. Thus, taxonomic assignments at the genus level, particularly within *Bradyrhizobium*, should be interpreted with caution.

In contrast, the relatively low abundance of *Bradyrhizobium* in both the BWL and triticale rhizosphere soils indicates a broader and more diverse microbial community outside the nodule niche. The similar microbial profiles of the BWL and triticale rhizosphere soils, particularly the dominant roles of Actinobacteriota and Proteobacteria, suggest that despite differences in plant physiology, both crops share a common foundation in their root-associated microbial communities. This shared microbial composition likely reflects similar soil conditions, such as acidic pH and low nutrient availability, in which both crops can thrive across the study area. Furthermore, the presence of phyla such as Acidobacteriota, Chloroflexi, and Gemmatimonadota in both rhizosphere soils indicates that these groups are well adapted to the local soil environment and may contribute to essential processes such as carbon cycling and phosphate metabolism (Hug et al., 2013; Kielak et al., 2016).

The enrichment of *Nocardioides* in the BWL rhizosphere soil (Figure 3) has been reported in literature as linked to phosphorus solubilization, suggesting possible functional roles that require experimental confirmation. In addition, the enrichment of *Sphingomonas* in both BWL and triticale rhizosphere soils highlights its potential as a plant growth-promoting microbe, as described in previous studies (Lombardino et al., 2022), though such functions were not directly validated in this study.

The observed distribution of ASVs (Figure 4) indicates strong niche partitioning among the different plant-associated environments. The high number of unique ASVs in the BWL and triticale rhizosphere soil samples suggests that the rhizosphere soil microbial communities are strongly influenced by plant-specific root exudates and architecture, leading to distinct microbial assemblages in each crop. Conversely, the considerably lower ASV richness in BWL nodules reflects the highly selective nature of the nodule environment, which is dominated by nitrogen-fixing symbionts such as *Bradyrhizobium* (Jaiswal and Dakora, 2019). This selective enrichment is critical for optimizing nitrogen fixation and overall plant health.

Moreover, the limited overlap of ASVs between nodules and rhizosphere soils, as well as the relatively small number of ASVs shared across all three environments (183), underscores the distinct ecological functions of these niches. This limited intersection highlights the strong niche differentiation and selective pressures exerted by plant species and tissue types on microbial community assembly. The small shared core underscores the specialized nature of the nodule microbiome compared to the more diverse and distinct rhizosphere soil communities. Unique ASVs found exclusively in each sample type further emphasize the adaptation of microbial taxa to specific plant-associated habitats, reflecting functional specialization that may contribute to the respective roles of nodules and rhizospheres in nutrient cycling and plant health. While the rhizosphere soils of both crops share a common microbial core that likely supports soil health and resilience (Berendsen et al., 2012; Dilla-Ermita et al., 2021; Abdullaeva et al., 2022), the nodule microbiome is specialized for symbiosis and nutrient exchange. These findings highlight that plant tissues impose specific selective pressures, shaping the microbial community composition in ways that optimize key functions such as nitrogen fixation in nodules and OM decomposition in the rhizosphere soil (Berg, 2009; Philippot et al., 2013).

As presented in Table 2, the greater microbial diversity in the rhizosphere soils than in the nodules suggests that the rhizosphere provides a more heterogeneous habitat, which is likely influenced by diverse root exudates and the dynamic soil environment. This heterogeneity may increase functional redundancy, contributing to more resilient nutrient cycling and pathogen suppression (Canarini et al., 2019). This finding is also consistent with previous reports indicating that the rhizosphere, as an interface between plant roots and soil, supports more diverse and complex microbial communities due to the heterogeneous availability of root exudates and soil resources (Philippot et al., 2013).

However, among the rhizosphere soil samples, the triticale rhizosphere soil from Kessa presented the highest microbial richness, and numerically, the triticale rhizosphere soils presented the highest microbial richness across all the sites except at Gumbla. This finding suggests a potentially more conducive root environment for microbial colonization than BWL. The numerical advantage observed in the triticale rhizosphere may be attributed to differences in root architecture or exudate composition that facilitate the recruitment of a broader range of microbial taxa (Marschner et al., 2004). This observation is consistent with ecological principles, where different plant tissues exert unique selective pressures, favoring microbial taxa with specialized functions (Berg, 2009; Philippot et al., 2013). The numerical advantage observed in triticale rhizosphere microbial diversity may be influenced by fertilization and repeated tillage practices applied only to triticale fields, which affect soil physicochemical properties and microbial communities. Additionally, the term “microbiome” used to describe nodule-associated communities refers here to the internal endophytic community, as nodules were surface-sterilized prior to DNA extraction. However, as no imprint test or equivalent validation was performed, residual epiphytic microbes cannot be fully excluded, and this limitation should be considered when interpreting the nodule microbiome.

### 4.3 Edaphic factors influencing microbial diversity and community structure

The strong positive correlation between microbial diversity and OM in triticale rhizosphere soil (Table 4) is consistent with previous studies that OM is positively associated with microbial richness, likely due to its role as a carbon and nutrients source (Chen et al., 2023). This finding supports the interpretation that OM is correlated with microbial diversity, potentially creating favorable microhabitats that sustain diverse microbial taxa (van der Heijden et al., 2008).

The positive correlation between TN and microbial richness in the triticale rhizosphere soil indicates a statistical association between nitrogen availability and microbial community structure in cereal-based agricultural soils. Previous studies have reported that nitrogen enrichment is correlated with diverse microbial populations, likely due to its role in nutrient cycling processes, such as nitrogen fixation and OM decomposition (Knežević et al., 2022). Taken together, the stronger correlations between microbial diversity and soil parameters such as OM and TN in triticale rhizosphere soils may reflect combined effect of plant species and fertilizer application, underscoring the influence of both biotic and management factors on microbial community structure. However, the lack of significant correlations in the BWL rhizosphere suggests that nitrogen related correlations may be crop-dependent and influenced by root exudates or plant-microbe interactions.

This study also revealed that the OC to clay ratio, AvP, and clay content significantly influenced the microbial composition across sites and sample types, as shown by the PCoA and dbRDA results (Figures 5, 6; Table 6). Together, these factors explained 8.06% of the total variance in microbial structure. As shown in Figure 6, the first two axes (CAP1 and CAP2) together explained more than 79% of the constrained variance (>6.4% of total variance) in the microbial structure. The role of soil properties, particularly nutrient availability and texture, is widely recognized as a determinant of microbial community dynamics (Lauber et al., 2008; Fierer, 2017).

The SHI, which reflects the ratio of OC to clay, emerged as statistically influential explanatory variable (*F* = 28%, *p* = 0.006), particularly separating communities along CAP1. Sites with relatively high SHI values, notably Kessa and Sawsa, especially in triticale rhizosphere soil samples, presented distinct microbial assemblages. This suggests that organic-rich soils provide increased carbon availability, supporting more specialized or abundant microbial taxa. Consistent with these findings, previous studies reported that OM contributes to the development of favorable microhabitats, which in turn sustain diverse microbial taxa (Bardgett and van der Putten, 2014; van der Heijden et al., 2008; Chen et al., 2023). Similarly, AvP (*F* = 26.7%, *p* = 0.016) was positively correlated with community shifts along both CAP1 and CAP2, with elevated phosphorus levels in Akana and Gumbla coinciding with unique microbial clusters, which might be due to the promotion of fast-growing microbes responsive to nutrient inputs. Phosphorus availability can directly influence the abundance of phosphate-solubilizing bacteria (Jiang et al., 2017). Clay content (*p* = 0.049) was also significantly associated with microbial structure, particularly contributing to tighter community clustering at clay-rich sites such as Debrekelmua and Amare, possibly due to improved moisture retention and physical protection of microbial habitats.

Notably, the sample type further controlled the influence of these environmental factors. As seen in the dbRDA plots (Figure 6), nodule samples clustered more tightly and consistently across sites, indicating that their microbial communities are less influenced by environmental variation and more strongly governed by host selection mechanisms. In contrast, rhizosphere soil samples from BWL and triticale were more widely dispersed, particularly along axes correlated with SHI (OC/clay) and AvP, suggesting that soil properties exert greater control over microbial assembly in the rhizosphere soil. Although SHI, AvP, and clay content were statistically significant drivers of microbial community composition, these factors together explained only a small proportion (8.06%) of the total variance. This indicates that most variations remain unexplained and another unmeasured variable likely play major roles. This is consistent with findings in other agroecosystems (Zhou et al., 2020; Jing et al., 2023), where additional factors such as microclimatic variations, soil moisture heterogeneity, plant root exudate profiles, and microbial interactions have been shown to substantially influence microbial community structure.

Overall, these findings demonstrate that a combination of host-associated and edaphic factors governs microbial diversity and community assembly. While this study provides valuable insights into microbial community composition and diversity, we recognize that functional validation is still needed to confirm the ecological roles of key taxa. Future work should incorporate approaches such as quantitative PCR (qPCR) of functional genes related to nitrogen fixation and nutrient cycling, as well as enzyme activity assays under field conditions. These complementary analyses would provide direct evidence of microbial functions and strengthen the link between community structure and ecosystem processes, thereby enhancing the practical relevance of microbiome management strategies in marginal soils.

### 4.4 Limitation and future direction

Several limitations should be noted. First, the comparison between BWL and triticale rhizospheres is confounded by differing agronomic practices (fertilization and tillage), which may influence microbial communities independently of plant species. Second, no agronomic outcomes (e.g., yield, nitrogen fixation efficiency) were measured, limiting direct applied relevance. Third, functional interpretations (nitrogen fixation, phosphorus solubilization, plant growth promotion) are based solely on taxonomic assignment and lack direct validation (e.g., via *nifH* or *phoD* qPCR or enzymatic assays). Fourth, 16S rRNA gene sequencing provides broad taxonomic profiling but has limited resolution for distinguishing closely related rhizobial species; therefore, taxonomic assignments at the genus level, particularly within *Bradyrhizobium*, are interpreted with caution. Fifth, network or co-occurrence analyses were not performed, which could have provided deeper ecological insight. Sixth, plant genotype–microbiome interactions were not assessed, despite claims of plant-driven selection. Future studies should standardize agronomic variables, include agronomic and functional measurements, apply network analyses, incorporate housekeeping genes (e.g., recA, glnII, atpD, or rpoB to enhance rhizobia taxonomic resolution and use controlled genetic backgrounds to disentangle plant genotype effects.

## 5 Conclusion

This study highlighted distinct microbial communities associated with BWL root nodules and the rhizosphere soils of BWL and neighboring triticale in acidic soils of northwestern Ethiopia. The specialized enrichment of nitrogen-fixing rhizobia (*Bradyrhizobium*) within BWL nodules implies the critical role of symbiotic associations in supporting crop nitrogen requirements under challenging soil conditions. Moreover, the rhizosphere soils of BWL and triticale harbor diverse microbial communities shaped by plant species and soil characteristics, with OC/clay, phosphorus availability, and clay content emerging as drivers of microbial community structure. The findings emphasize the complex interaction between plant hosts and soil properties in shaping microbial assemblages that contribute to soil health and nutrient cycling. However, while these findings advance our understanding of plant-microbe interactions in soil, it is acknowledged that differences in fertilization and tillage practices between crops constitute fundamental confounding factors. The ecological functions of key microbial taxa remain to be experimentally validated. Future research should therefore integrate qPCR of functional genes involved in nitrogen fixation and nutrient cycling, and enzyme activity assays under field conditions. Such functional validation will be critical to confirm the roles of microbial communities and to translate these insights into effective microbiome management strategies for soil health improvement.

## Data Availability

The datasets are deposited in the NCBI Sequence Read Archive under BioProject accession PRJNA1460899, which corresponds to the official record available at: http://www.ncbi.nlm.nih.gov/bioproject/1460899.

## References

[ref1] Abdullaeva Y. Ratering S. Ambika Manirajan B. Rosado-Porto D. Schnell S. Cardinale M. (2022). Domestication impacts the wheat-associated microbiota and the rhizosphere colonization by seed- and soil-originated microbiomes, across different fields. Front. Plant Sci. 12:806915. doi: 10.3389/fpls.2021.806915, 35095978 PMC8789879

[ref2] Agudelo J. Miller A. W. (2025). Impact of study design, contamination, and data characteristics on results and interpretation of microbiome studies. mSystems 10, e00408–e00425. doi: 10.1128/msystems.00408-25, 40767516 PMC12456016

[ref3] Alawiye T. T. Babalola O. O. (2019). Bacterial diversity and community structure in typical plant rhizosphere. Diversity 11:179. doi: 10.3390/d11100179

[ref4] Bardgett R. D. Van Der Putten W. H. (2014). Belowground biodiversity and ecosystem functioning. Nature 515, 505–511. doi: 10.1038/nature13855, 25428498

[ref15] Benesty J. Chen J. Huang Y. Cohen I. (2009). Pearson correlation coefficient. Noise Reduction in Speech Processing Springer Topics in Signal Processing 2, 1–4. doi: 10.1007/978-3-642-00296-0_5

[ref5] Berendsen R. L. Pieterse C. M. J. Bakker P. A. H. M. (2012). The rhizosphere microbiome and plant health. Trends Plant Sci. 17, 478–486. doi: 10.1016/j.tplants.2012.04.001, 22564542

[ref6] Berg G. (2009). Plant–microbe interactions promoting plant growth and health: perspectives for controlled use of microorganisms in agriculture. Appl. Microbiol. Biotechnol. 84, 11–18. doi: 10.1007/s00253-009-2092-7, 19568745

[ref7] Bouyoucos G. J. (1962). Hydrometer method improved for making particle size analyses of soils 1. Agron. J. 54, 464–465. doi: 10.2134/agronj1962.00021962005400050028x

[ref8] Bremner J. (1965). Total nitrogen. Methods of Soil Analysis: Part 2 Chemical and Microbiological Properties, 9, 1149–1178, Total Nitrogen, doi: 10.2134/agronmonogr9.2.c32.

[ref9] Cai A. Tang S. Waqas M. A. Wang B. Tian D. Zhang Y. . (2023). Magnitude, direction, and drivers of rhizosphere effect on soil nitrogen and phosphorus in global agroecosystem. Int. Soil Water Conserv. Res. 11, 482–493. doi: 10.1016/j.iswcr.2022.07.004

[ref10] Callahan B. J. Mcmurdie P. J. Rosen M. J. Han A. W. Johnson A. J. A. Holmes S. P. (2016). DADA2: high-resolution sample inference from Illumina amplicon data. Nat. Methods 13, 581–583. doi: 10.1038/nmeth.3869, 27214047 PMC4927377

[ref11] Canarini A. Kaiser C. Merchant A. Richter A. Wanek W. (2019). Root exudation of primary metabolites: mechanisms and their roles in plant responses to environmental stimuli. Frontiers. Plant Sci. 10:157. doi: 10.3389/fpls.2019.00157, 30881364 PMC6407669

[ref12] Chaddad Z. Lamrabet M. Bennis M. Kaddouri K. Alami S. Bouhnik O. . (2024). “Nitrogen-fixing Bradyrhizobium spp. as plant growth-promoting Bacteria to improve soil quality and plant tolerance to biotic and abiotic stresses,” in Soil Bacteria: Biofertilization and Soil Health, eds. Dheeman S. Islam M. T. Egamberdieva D. Siddiqui M. N. (Singapore: Springer Nature Singapore).

[ref13] Chen H. (2018). VennDiagram: generate high-resolution Venn and Euler plots. R Package Version 1:1. Available online at: https://CRAN.Rproject.org/package=VennDiagram

[ref14] Chen W. Hu H. Heal K. Sohi S. Tigabu M. Qiu W. . (2023). Linking microbial decomposition to dissolved organic Matter composition in the revegetation of the red soil Erosion area. Forests 14:270. doi: 10.3390/f14020270

[ref16] Dakora F. D. Phillips D. A. (2002). Root exudates as mediators of mineral acquisition in low-nutrient environments. Plant Soil 245, 35–47. doi: 10.1023/A:1020809400075

[ref17] Das P. P. Singh K. R. Nagpure G. Mansoori A. Singh R. P. Ghazi I. A. . (2022). Plant-soil-microbes: a tripartite interaction for nutrient acquisition and better plant growth for sustainable agricultural practices. Environ. Res. 214:113821. doi: 10.1016/j.envres.2022.113821, 35810815

[ref18] Dilla-Ermita C. J. Lewis R. W. Sullivan T. S. Hulbert S. H. (2021). Wheat genotype-specific recruitment of rhizosphere bacterial microbiota under controlled environments. Front. Plant Sci. 12:718264. doi: 10.3389/fpls.2021.718264, 34925393 PMC8671755

[ref19] Fierer N. (2017). Embracing the unknown: disentangling the complexities of the soil microbiome. Nat. Rev. Microbiol. 15, 579–590. doi: 10.1038/nrmicro.2017.87, 28824177

[ref20] Herlemann D. P. R. Labrenz M. Jürgens K. Bertilsson S. Waniek J. J. Andersson A. F. (2011). Transitions in bacterial communities along the 2000 km salinity gradient of the Baltic Sea. ISME J. 5, 1571–1579. doi: 10.1038/ismej.2011.41, 21472016 PMC3176514

[ref21] Howieson J. Dilworth M. (2016). Working With Rhizobia. Australia: Australian Centre for International Agricultural Research Canberra.

[ref22] Hug L. A. Castelle C. J. Wrighton K. C. Thomas B. C. Sharon I. Frischkorn K. R. . (2013). Community genomic analyses constrain the distribution of metabolic traits across the Chloroflexi phylum and indicate roles in sediment carbon cycling. Microbiome 1:22. doi: 10.1186/2049-2618-1-22, 24450983 PMC3971608

[ref23] I-Hsuan L. (2021). 16S rDNA amplicon sequencing analysis using R. ycl6.github.io. Available online at: https://ycl6.github.io/16S-Demo/index.html

[ref24] Jaiswal S. K. Dakora F. D. (2019). Widespread distribution of highly adapted *Bradyrhizobium* species Nodulating diverse legumes in Africa. Front. Microbiol. 10:310. doi: 10.3389/fmicb.2019.00310, 30853952 PMC6395442

[ref25] Jiang Y. Liu M. Zhang J. Chen Y. Chen X. Chen L. . (2017). Nematode grazing promotes bacterial community dynamics in soil at the aggregate level. ISME J. 11, 2705–2717. doi: 10.1038/ismej.2017.120, 28742069 PMC5702727

[ref26] Jing H. Wang H. Wang G. Liu G. Cheng Y. (2023). The mechanism effects of root exudate on microbial community of rhizosphere soil of tree, shrub, and grass in forest ecosystem under N deposition. ISME Communications 3:120. doi: 10.1038/s43705-023-00322-9, 37985715 PMC10662252

[ref27] Johannes A. Matter A. Schulin R. Weisskopf P. Baveye P. C. Boivin P. (2017). Optimal organic carbon values for soil structure quality of arable soils. Does clay content matter? Geoderma 302, 14–21. doi: 10.1016/j.geoderma.2017.04.021

[ref28] Kandlikar G. Gold Z. Cowen M. Meyer R. Freise A. Kraft N. . (2018). Ranacapa: an R package and shiny web app to explore environmental DNA data with exploratory statistics and interactive visualizations [version 1; peer review: 1 approved, 2 approved with reservations]. F1000Res. 7:1734. doi: 10.12688/f1000research.16680.1 30613396 PMC6305237

[ref29] Kefale B. Fanta S. W. Satheesh N. (2022). Review on nutritional, anti nutritional content and effect of processing on anti nutritional content of lupine in Ethiopia. Eur. J. Agric. For. Res. 10, 56–81. doi: 10.37745/ejafr.2013

[ref30] Kielak A. M. Barreto C. C. Kowalchuk G. A. Van Veen J. A. Kuramae E. E. (2016). The ecology of Acidobacteria: moving beyond genes and genomes. Front. Microbiol. 7:744. doi: 10.3389/fmicb.2016.00744, 27303369 PMC4885859

[ref31] Knežević M. Buntić A. Delić D. Stajković-Srbinović O. (2022). “Root nodule bacteria-rhizobia: exploring the beneficial effects on non-legume plant growth,” in Nitrogen Fixing Bacteria: Sustainable Growth of Non-Legumes, eds. Maheshwari D. K. Dobhal R. Dheeman S. (Singapore: Springer Nature Singapore).

[ref32] Kui L. Chen B. Chen J. Sharifi R. Dong Y. Zhang Z. . (2021). A comparative analysis on the structure and function of the Panax notoginseng rhizosphere microbiome. Front. Microbiol. 12:673512. doi: 10.3389/fmicb.2021.673512, 34177857 PMC8219928

[ref33] Lahti L. Shetty S. Turaga N. Obenchain V. Salojärvi J. Gilmore R. (2017). Tools for microbiome analysis in R. Version Available online at: http://microbiome.github.com/microbiome

[ref34] Lauber C. L. Strickland M. S. Bradford M. A. Fierer N. (2008). The influence of soil properties on the structure of bacterial and fungal communities across land-use types. Soil Biol. Biochem. 40, 2407–2415. doi: 10.1016/j.soilbio.2008.05.021

[ref35] Li Y. L. Fan X. R. Shen Q. R. (2008). The relationship between rhizosphere nitrification and nitrogen-use efficiency in rice plants. Plant Cell Environ. 31, 73–85. doi: 10.1111/j.1365-3040.2007.01737.x, 17944815

[ref36] Lombardino J. Bijlani S. Singh N. K. Wood J. M. Barker R. Gilroy S. . (2022). Genomic characterization of potential plant growth-promoting features of *Sphingomonas* strains isolated from the international Space Station. Microbiol. Spectrum 10:e0199421. doi: 10.1128/spectrum.01994-21, 35019675 PMC8754149

[ref37] Love M. Huber W. Anders S. (2014). Differential analysis of count data–the DESeq2. Genome Biol. 15:550. doi: 10.1186/s13059-014-0550-8 25516281 PMC4302049

[ref38] Marschner P. Crowley D. Yang C. H. (2004). Development of specific rhizosphere bacterial communities in relation to plant species, nutrition and soil type. Plant Soil 261, 199–208. doi: 10.1023/b:plso.0000035569.80747.c5

[ref39] Martin M. (2011). Cutadapt removes adapter sequences from high-throughput sequencing reads. EMBnet. Journal 17, 10–12. doi: 10.14806/ej.17.1.200

[ref40] Mcmurdie P. J. Holmes S. (2013). Phyloseq: an R package for reproducible interactive analysis and graphics of microbiome census data. PLoS One 8:e61217. doi: 10.1371/journal.pone.0061217, 23630581 PMC3632530

[ref41] Mousavi S. A. Gao Y. Penttinen P. Frostegård Å. Paulin L. Lindström K. (2022). Using amplicon sequencing of rpoB for identification of inoculant rhizobia from peanut nodules. Lett. Appl. Microbiol. 74, 204–211. doi: 10.1111/lam.13599, 34753197

[ref42] Ogle D. H. Doll J. C. Wheeler A. P. Dinno A. (2023). FSA: simple fisheries stock assessment methods. R Package Version 0.9 4. doi: 10.32614/CRAN.package.FSA

[ref43] Oksanen J. Blanchet F. G. Kindt R. Legendre P. Minchin P. R. O’hara R. . (2013). Package ‘vegan’. Community Ecology Package, Version 2, 1–295. doi: 10.32614/CRAN.package.vegan

[ref44] Olsen S. R. (1954). Estimation of Available Phosphorus in Soils by Extraction With Sodium Bicarbonate. Washington, D.C., USA: US Department of Agriculture.

[ref45] Philippot L. Raaijmakers J. M. Lemanceau P. Van Der Putten W. H. (2013). Going back to the roots: the microbial ecology of the rhizosphere. Nat. Rev. Microbiol. 11, 789–799. doi: 10.1038/nrmicro3109, 24056930

[ref46] Prout J. M. Shepherd K. D. Mcgrath S. P. Kirk G. J. Haefele S. M. (2021). What is a good level of soil organic matter? An index based on organic carbon to clay ratio. Eur. J. Soil Sci. 72, 2493–2503. doi: 10.1111/ejss.13012

[ref47] Quast C. Pruesse E. Yilmaz P. Gerken J. Schweer T. Yarza P. . (2012). The SILVA ribosomal RNA gene database project: improved data processing and web-based tools. Nucleic Acids Res. 41, D590–D596. doi: 10.1093/nar/gks1219, 23193283 PMC3531112

[ref48] Sahlemedhin S. Taye B. (2000). Procedure for soil and plant analysis. National soil research Centre, Ethiopian agricultural research organization, Addis Ababa, Ethiopia. Soil Sci. Soc. Am. J. 70:287.

[ref49] Sauzet O. Johannes A. Deluz C. Dupla X. Matter A. Baveye P. C. . (2024). The organic carbon-to-clay ratio as an indicator of soil structure vulnerability, a metric focused on the condition of soil structure. Soil Use Manag. 40:e13060. doi: 10.1111/sum.13060

[ref50] Seddaiu G. Porcu G. Ledda L. Roggero P. P. Agnelli A. Corti G. (2013). Soil organic matter content and composition as influenced by soil management in a semi-arid Mediterranean agro-silvo-pastoral system. Agric. Ecosyst. Environ. 167, 1–11. doi: 10.1016/j.agee.2013.01.002

[ref51] Sharma D. Gahtyari N. C. Chhabra R. Kumar D. (2020). Role of microbes in improving plant growth and soil health for sustainable agriculture. In: Adv. Plant Microbiome Sustain. Agric.: Divers. Biotechnol. Appl. Singapore: Microorganisms for Sustainability, Springer 19, 207–256. doi: 10.1007/978-981-15-3208-5_9

[ref52] Sharma S. K. Ramesh A. Sharma M. P. Joshi O. P. Govaerts B. Steenwerth K. L. . (2011). “Microbial community structure and diversity as indicators for evaluating soil quality,” in Biodiversity, Biofuels, Agroforestry and Conservation Agriculture, ed. Lichtfouse E. (Dordrecht: Springer Netherlands).

[ref53] Simmons T. Caddell D. F. Deng S. Coleman-Derr D. (2018). Exploring the root microbiome: extracting bacterial community data from the soil, rhizosphere, and root endosphere. J. Vis. Exp. doi: 10.3791/57561, 29782021 PMC6101100

[ref54] Sohn S. I. Ahn J. H. Pandian S. Oh Y. J. Shin E. K. Kang H. J. . (2021). Dynamics of bacterial community structure in the rhizosphere and root nodule of soybean: impacts of growth stages and varieties. Int. J. Mol. Sci. 22:5577. doi: 10.3390/ijms22115577, 34070397 PMC8197538

[ref55] Sun L. Ataka M. Kominami Y. Yoshimura K. Kitayama K. (2021). A constant microbial C/N ratio mediates the microbial nitrogen mineralization induced by root exudation among four co-existing canopy species. Rhizosphere 17:100317. doi: 10.1016/j.rhisph.2021.100317

[ref56] R Core Team (2024). R: A Language and Environment for Statistical Computing [Online]. Vienna, Austria: R Foundation for Statistical Computing.

[ref57] Thijs S. Op De Beeck M. Beckers B. Truyens S. Stevens V. Van Hamme J. D. . (2017). Comparative evaluation of four bacteria-specific primer pairs for 16S rRNA gene surveys. Front. Microbiol. 8:494. doi: 10.3389/fmicb.2017.00494, 28400755 PMC5368227

[ref58] Tiziani R. Mimmo T. Valentinuzzi F. Pii Y. Celletti S. Cesco S. (2021). Corrigendum: root handling affects carboxylates exudation and phosphate uptake of white Lupin roots. Front. Plant Sci. 12:681263. doi: 10.3389/fpls.2021.681263, 33968123 PMC8101549

[ref59] Tukey J. W. (1949). Comparing individual means in the analysis of variance. Biometrics 5, 99–114. doi: 10.2307/3001913 18151955

[ref60] Van Der Heijden M. G. Bardgett R. D. Van Straalen N. M. (2008). The unseen majority: soil microbes as drivers of plant diversity and productivity in terrestrial ecosystems. Ecol. Lett. 11, 296–310. doi: 10.1111/j.1461-0248.2007.01139.x, 18047587

[ref61] Walkley A. Black I. A. (1934). An examination of the Degtjareff method for determining soil organic matter, and a proposed modification of the chromic acid titration method. Soil Sci. 37, 29–38. doi: 10.1097/00010694-193401000-00003

[ref62] Wang R. Sun C. Cai S. Liu F. Xie H. Xiong Q. (2023). Research progress in crop root biology and nitrogen uptake and use, with emphasis on cereal crops. Agron. 13:1678. doi: 10.3390/agronomy13071678

[ref63] Wickham H. (2016). ggplot2: Elegant Graphics for Data Analysis. Springer-Verlag New York, USA.

[ref64] Wickham H. François R. Henry L. Müller K. Vaughan D. (2023). dplyr: a grammar of data manipulation. R package version 1.1. 2. Computer software.

[ref65] Yeheyis L. Kijora C. Wink M. Peters K. J. (2011). Effect of a traditional processing method on the chemical composition of local white Lupin (*Lupinus albus* L.) seed in north-western Ethiopia. Zeitschrift für Naturforschung C 66, 403–408. doi: 10.1515/znc-2011-7-812, 21950165

[ref66] Zhou Y. Coventry D. R. Gupta V. V. S. R. Fuentes D. Merchant A. Kaiser B. N. . (2020). The preceding root system drives the composition and function of the rhizosphere microbiome. Genome Biol. 21:89. doi: 10.1186/s13059-020-01999-0, 32252812 PMC7137527

