# Supplementary material for: Microbial community composition and diversity in nodules and rhizosphere soil of bitter white lupine (Lupinus albus L.) and rhizosphere soil of triticale (×Triticosecale Wittmack)
Source: Front Microbiol. 2026 Jun 24;17:1810398. doi: 10.3389/fmicb.2026.1810398 (PMC13353089; doi:10.3389/fmicb.2026.1810398)
Supplement: Supplementary file 2 [file Table_2.docx]

Supplementary Material

# Supplementary Tables

**Supplementary Table 1.** Two‑way ANOVA results for soil physicochemical properties (pH, OC, OM, TN, CN, AvP, CEC, Sand, Clay, Silt, SHI) as a function of Sample_type (BWL_RS vs. Triticale_RS) and Sampling_site (six sites: Akana, Kessa, Amare, Debre Kelmua, Gumbla, Sawsa) and their interaction

| **Soil property** | **Factor** | **Sum Square** | **Mean Square** | **F value** | **p value** |
| --- | --- | --- | --- | --- | --- |
|  | Sample_type | 0.407 | 0.407 | 4.267 | 0.050 * |
| pH | Sampling_site | 1.936 | 0.387 | 4.064 | 0.008 ** |
|  | Sample_type × Sampling_site | 0.309 | 0.062 | 0.649 | 0.665 |
|  | Sample_type | 0.530 | 0.535 | 1.122 | 0.3 |
| OC | Sampling_site | 43.810 | 8.761 | 18.382 | 1.64e-07 *** |
|  | Sample_type × Sampling_site | 1.280 | 0.257 | 0.538 | 0.745 |
|  | Sample_type | 1.590 | 1.589 | 1.122 | 0.3 |
| OM | Sampling_site | 130.200 | 26.041 | 18.382 | 1.64e-07 *** |
|  | Sample_type × Sampling_site | 3.810 | 0.762 | 0.538 | 0.745 |
|  | Sample_type | 0.003 | 0.003 | 1.8 | 0.192 |
| TN | Sampling_site | 0.114 | 0.023 | 16.353 | 4.75e-07 *** |
|  | Sample_type × Sampling_site | 0.010 | 0.002 | 1.487 | 0.231 |
|  | Sample_type | 0.310 | 0.310 | 0.045 | 0.833 |
| CN | Sampling_site | 254.690 | 50.940 | 7.543 | 2.23e-04 *** |
|  | Sample_type × Sampling_site | 26.990 | 5.400 | 0.799 | 0.561 |
|  | Sample_type | 337.000 | 337.000 | 3.167 | 0.088 . |
| AvP | Sampling_site | 999.800 | 200.000 | 1.879 | 0.136 |
|  | Sample_type × Sampling_site | 234.400 | 46.900 | 0.441 | 0.816 |
|  | Sample_type | 32.900 | 32.870 | 2.376 | 0.136 |
| CEC | Sampling_site | 379.600 | 75.920 | 5.488 | 0.002 ** |
|  | Sample_type × Sampling_site | 198.700 | 39.740 | 2.873 | 0.036 * |
|  | Sample_type | 1.000 | 1.000 | 0.054 | 0.818 |
| Sand | Sampling_site | 1489.300 | 297.870 | 16.125 | 5.39e-07 *** |
|  | Sample_type × Sampling_site | 94.300 | 18.870 | 1.021 | 0.427 |
|  | Sample_type | 2.000 | 2.000 | 0.044 | 0.836 |
| Clay | Sampling_site | 2724.500 | 544.900 | 11.963 | 6.95e-06 *** |
|  | Sample_type × Sampling_site | 94.200 | 18.800 | 0.414 | 0.835 |
|  | Sample_type | 0.170 | 0.170 | 0.02 | 0.889 |
| Silt | Sampling_site | 232.030 | 46.410 | 5.299 | 0.002 ** |
|  | Sample_type × Sampling_site | 61.200 | 12.240 | 1.398 | 0.261 |
|  | Sample_type | 0.003 | 0.003 | 1.833 | 0.188 |
| SHI | Sampling_site | 0.144 | 0.029 | 19.712 | 8.55e-08 *** |
|  | Sample_type × Sampling_site | 0.004 | 0.001 | 0.535 | 0.748 |

*Sample_type (df=1) = BWL_RS and Triticale_RS; Sampling_site (df=5) = Akana, Kessa, Amare, Debrekelmua, Gumbla and Sawsa; Sample_type × Sampling_site= the interaction effect; SHI= soil health indicator. The significance codes are 0 ‘***’ 0.001 ‘**’ 0.01 ‘*’ 0.05 ‘.’ 0.1 ‘ ’ 1*

**Supplementary Table 2.** Summary of sequencing read counts and ASV richness per sample at each stage of the DADA2 pipeline

| **Sample_ID** | **Raw_Reads** | **Filtered_Reads** | **Chimera_Free_Reads** | **ASV_Counts** | **Sample_ID** | **Raw_Reads** | **Filtered_Reads** | **Chimera_Free_Reads** | **ASV_Counts** |
| --- | --- | --- | --- | --- | --- | --- | --- | --- | --- |
| BHaN1 | 104398 | 79790 | 29776 | 160 | MDaN1 | 97357 | 74970 | 67048 | 72 |
| BHaN2 | 114029 | 88464 | 39903 | 96 | MDaN2 | 94621 | 73257 | 67885 | 77 |
| BHaN3 | 94473 | 72477 | 39447 | 89 | MDaN3 | 124486 | 95925 | 80854 | 170 |
| BHbN1 | 92826 | 70934 | 39570 | 114 | MDbN1 | 147035 | 113270 | 106482 | 59 |
| BHbN2 | 84037 | 65235 | 46272 | 67 | MDbN2 | 108835 | 84087 | 78133 | 52 |
| BHbN3 | 145555 | 112341 | 110277 | 45 | MDbN3 | 156122 | 119761 | 117927 | 52 |
| BHcN1 | 93827 | 71693 | 33988 | 113 | MDcN1 | 84049 | 65569 | 28884 | 159 |
| BHcN2 | 80276 | 59893 | 30763 | 73 | MDcN2 | 120180 | 92160 | 71533 | 149 |
| BHcN3 | 84945 | 64325 | 43120 | 166 | MDcN3 | 74078 | 56051 | 34241 | 89 |
| BHLa | 96929 | 71803 | 28661 | 1411 | MDLa | 71888 | 52795 | 19682 | 1144 |
| BHLb | 128021 | 94401 | 46462 | 1729 | MDLb | 87902 | 63351 | 30234 | 1369 |
| BHLc | 128332 | 94568 | 42812 | 1750 | MDLc | 86216 | 63023 | 24003 | 1278 |
| BHWa | 107410 | 79099 | 34217 | 1904 | MDWa | 86978 | 53027 | 24006 | 1378 |
| BHWb | 100728 | 74299 | 35025 | 1768 | MDWb | 94068 | 69837 | 25773 | 1343 |
| BHWc | 96936 | 71466 | 31827 | 1523 | MDWc | 70462 | 51709 | 17641 | 1130 |
| BKaN1 | 140126 | 107942 | 79262 | 102 | SGaN1 | 96185 | 72621 | 37506 | 257 |
| BKaN2 | 155743 | 119273 | 109258 | 30 | SGaN2 | 99463 | 77332 | 52856 | 66 |
| BKaN3 | 88926 | 68869 | 21613 | 107 | SGaN3 | 92211 | 70855 | 57490 | 69 |
| BKbN1 | 97514 | 75266 | 41231 | 120 | SGbN1 | 104983 | 81380 | 37126 | 165 |
| BKbN2 | 111709 | 86051 | 85207 | 17 | SGbN2 | 127643 | 98372 | 84416 | 29 |
| BKbN3 | 152040 | 118173 | 114792 | 35 | SGbN3 | 111813 | 87198 | 66717 | 33 |
| BKcN1 | 95864 | 73868 | 24431 | 182 | SGcN1 | 109849 | 84466 | 65933 | 97 |
| BKcN2 | 106658 | 81673 | 38547 | 192 | SGcN2 | 94528 | 73259 | 66344 | 97 |
| BKcN3 | 109438 | 83325 | 56389 | 126 | SGcN3 | 139386 | 108525 | 103595 | 93 |
| BKLa | 94891 | 69516 | 30647 | 1486 | SGLa | 107263 | 75758 | 25338 | 1511 |
| BKLb | 93136 | 68634 | 28880 | 1334 | SGLb | 114185 | 81518 | 28248 | 1628 |
| BKLc | 122374 | 90548 | 40638 | 1870 | SGLc | 95227 | 69681 | 20771 | 1366 |
| BKWa | 104208 | 77262 | 39603 | 2102 | SGWa | 79897 | 59521 | 17750 | 1335 |
| BKWb | 134007 | 99646 | 53517 | 2212 | SGWb | 76174 | 56276 | 17016 | 1249 |
| BKWc | 87465 | 65048 | 29764 | 1769 | SGWc | 97436 | 61768 | 32293 | 1730 |
| MAaN1 | 85785 | 66045 | 46104 | 87 | SSaN1 | 113363 | 86078 | 43298 | 162 |
| MAaN2 | 124070 | 95544 | 70735 | 151 | SSaN2 | 140474 | 108139 | 76824 | 132 |
| MAaN3 | 94479 | 72708 | 64589 | 87 | SSaN3 | 111405 | 85769 | 68902 | 89 |
| MAbN1 | 126506 | 96845 | 70670 | 222 | SSbN1 | 118666 | 92221 | 64845 | 85 |
| MAbN2 | 118649 | 91999 | 53766 | 136 | SSbN2 | 110014 | 84986 | 67063 | 101 |
| MAbN3 | 87265 | 67669 | 32685 | 110 | SSbN3 | 101444 | 78449 | 48841 | 63 |
| MAcN1 | 113308 | 87551 | 48348 | 87 | SScN1 | 125921 | 97533 | 57814 | 167 |
| MAcN2 | 104286 | 80839 | 34768 | 98 | SScN2 | 144449 | 111921 | 58330 | 123 |
| MAcN3 | 110200 | 85587 | 80084 | 51 | SScN3 | 149046 | 114372 | 69216 | 135 |
| MALa | 98510 | 73154 | 37387 | 1462 | SSLa | 109302 | 80955 | 37030 | 1027 |
| MALb | 77972 | 56946 | 27229 | 1258 | SSLb | 88956 | 65899 | 36075 | 1541 |
| MALc | 98405 | 72213 | 38368 | 1586 | SSLc | 86308 | 63447 | 33390 | 1303 |
| MAWa | 96073 | 66929 | 30919 | 1390 | SSWa | 95173 | 69882 | 33490 | 1533 |
| MAWb | 95142 | 70089 | 23121 | 1333 | SSWb | 98403 | 71995 | 34712 | 1573 |
| MAWc | 122154 | 88647 | 40641 | 2078 | SSWc | 86991 | 64792 | 30999 | 1632 |

*Sample_ID: unique sample identifier.* *The first letters in the Sample_ID column represent the name of the district (B= Banja, M= Machakel, S= Sekela) where this research was performed. The second letter indicates the sampling sites considered in this research (H= Akana, K= Kessa, A= Amare, D= Debrekelmua, G= Gumbla, and S= Sawsa). The third letter indicates either the Triticale field (W) or the BWL field (L). Lowercase letters (a, b, and c) represent replications within each sampling site. N1, N2, and N3 represent the nodules per plant sampled from each sampling replicate. Raw_Reads: number of raw paired‑end reads before filtering. Filtered_Reads: reads remaining after quality filtering (truncLen = c(260,200), maxEE = c(2,5), truncQ = 2). Chimera_Free_Reads: reads after chimera removal (consensus method). ASV_Counts: number of unique amplicon sequence variants (ASVs) detected in the sample after chimera removal. All samples were processed together; the minimum library size after chimera removal was 17,016 reads (sample SGWb).*

**Supplementary Table 3.** Analysis of variance (ANOVA) results for diversity indices using Aligned Rank Transform (ART)

| **Term** | **Df** | **Sum Square** | **F value** | **p value** | **η^2^p (%)** |
| --- | --- | --- | --- | --- | --- |
| **Observed Index** | | | | | |
| Sample_type | 2 | 44300.11 | 98.130 | 2.73E-21 | 73.16 |
| Sampling_site | 5 | 11442.33 | 4.117 | 0.002 | 22.23 |
| Sample_type × Sampling_site | 10 | 24970.76 | 5.399 | 6.38E-06 | 42.85 |
| **Shannon Index** | | | | | |
| Sample_type | 2 | 45060.11 | 106.464 | 3.11E-22 | 74.73 |
| Sampling_site | 5 | 7711.13 | 2.145 | 0.070 | 12.97 |
| Sample_type × Sampling_site | 10 | 6928.90 | 0.932 | 0.509 | 11.47 |
| **Simpson Index** | | | | | |
| Sample_type | 2 | 39131.78 | 67.177 | 3.45E-17 | 65.11 |
| Sampling_site | 5 | 10904.47 | 3.232 | 0.011 | 18.33 |
| Sample_type × Sampling_site | 10 | 9989.39 | 1.445 | 0.179 | 16.71 |

*This table presents the ART (aligned rank transform) ANOVA results for three diversity indices (observed, Shannon, and Simpson) across different sample types and sampling sites. The terms included in the model are as follows:*

*Sample_type: Represents different sample categories (e.g., BWL_RS, Nodule, and Triticale_RS).*

*Sampling_site: Different locations where samples were collected* (*Akana, Kessa, Amare, Debre Kelmua, Gumbla, Sawsa)*

*Sample_type × Sampling_site: Interaction effect between sample type and sampling site.*

*η^2^p (%): Partial eta squared, representing the effect size as a percentage of variance explained by each factor.*

**Supplementary Table 4.** List of ASVs detected in each sample, with read counts, relative abundances (%), and taxonomic assignments (Kingdom to Genus)

# Supplementary Figure


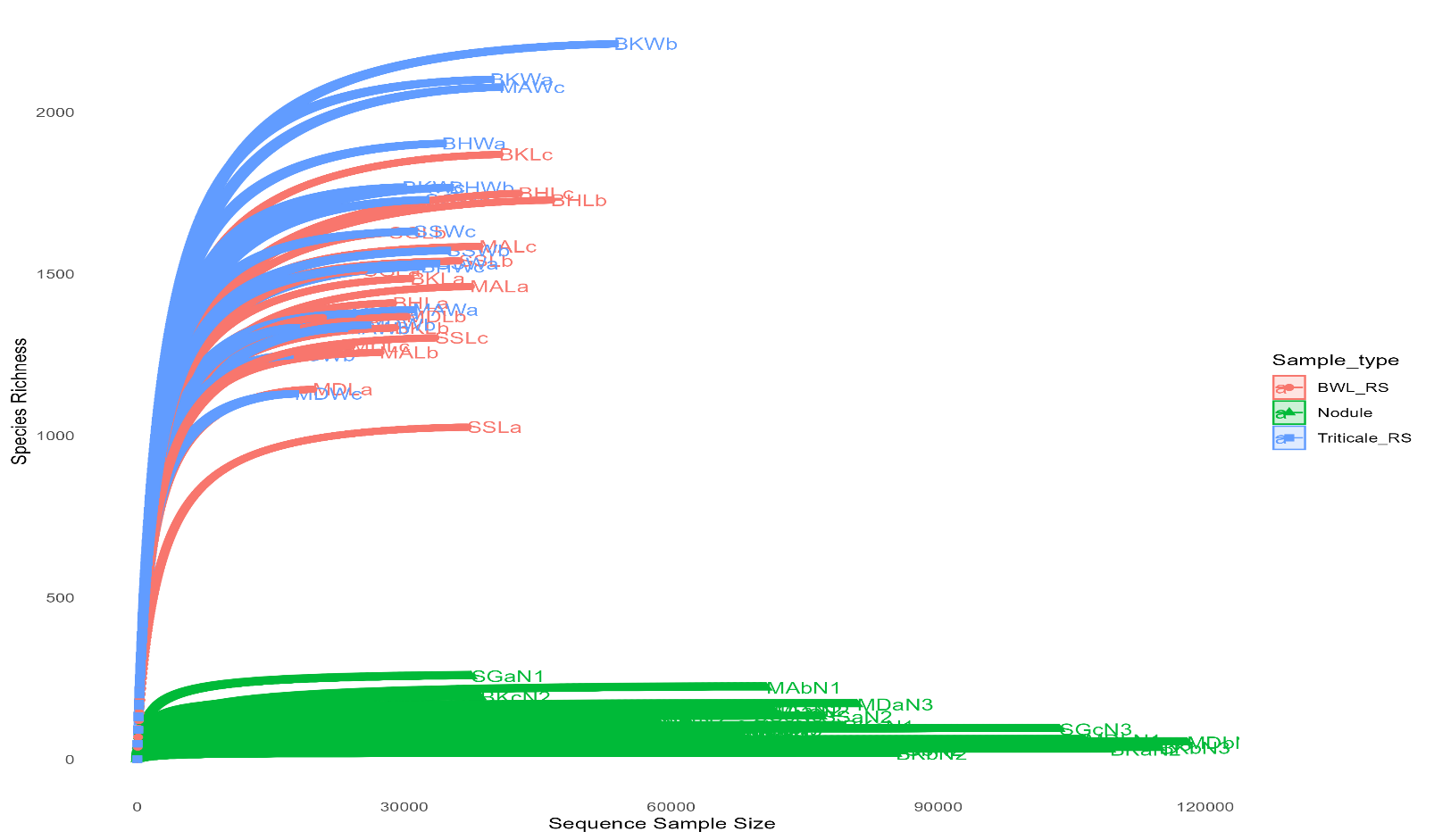


**Supplementary Figure 1.** Rarefaction curves based on the sequences of the V3-V4 region of the 16S rRNA gene from samples associated with BWL root nodules, BWL-RS and Triticale_RS (triticale rhizosphere soils)

*This figure shows the rarefaction curves generated for the 16S rRNA gene sequencing data, which visualize the sequencing depth and species richness across the three sample types: BWL root nodules, BWL rhizosphere soil (BWL-RS), and triticale rhizosphere soil (Triticale_RS). The x-axis represents the number of sequences, whereas the y-axis represents the number of observed amplicon sequence variants (ASVs). The rarefaction curves indicate that the sequencing depth was sufficient to capture the bacterial diversity in all sample types.*
